# High-precision solid catalysts for investigation of carbon nanotube synthesis and structure

**DOI:** 10.1126/sciadv.abb6010

**Published:** 2020-09-30

**Authors:** Xiao Zhang, Brian Graves, Michael De Volder, Wenming Yang, Tyler Johnson, Bo Wen, Wei Su, Robert Nishida, Sishen Xie, Adam Boies

**Affiliations:** 1 Department of Engineering, University of Cambridge, Cambridge CB2 1PZ, UK.; 2 School of Mechanical Engineering, University of Science and Technology Beijing, Beijing 100083, China.; 3 Department of Chemistry, University of Cambridge, Cambridge CB2 1EW, UK.; 4 Cambridge Graphene Centre, University of Cambridge, Cambridge CB3 0FA, UK.; 5 Institute of Physics, Chinese Academy of Sciences, P. O. Box 603, Haidian, Beijing 100190, China.

## Abstract

The direct growth of single-walled carbon nanotubes (SWCNTs) with narrow chiral distribution remains elusive despite substantial benefits in properties and applications. Nanoparticle catalysts are vital for SWCNT and more generally nanomaterial synthesis, but understanding their effect is limited. Solid catalysts show promise in achieving chirality-controlled growth, but poor size control and synthesis efficiency hampers advancement. Here, we demonstrate the first synthesis of refractory metal nanoparticles (W, Mo, and Re) with near-monodisperse sizes. High concentrations (*N* = 10^5^ to 10^7^ cm^−3^) of nanoparticles (diameter 1 to 5 nm) are produced and reduced in a single process, enabling SWCNT synthesis with controlled chiral angles of 19° ± 5°, demonstrating abundance >93%. These results confirm the interface thermodynamics and kinetic growth theory mechanism, which has been extended here to include temporal dependence of fast-growing chiralities. The solid catalysts are further shown effective via floating catalyst growth, offering efficient production possibilities.

## Teaser

Continuous uniform <2-nm refractory catalyst production is realized for selective chiral SWCNT growth.

## INTRODUCTION

Nanoparticle (NP) composition and size have a first-order impact on catalyst selectivity and efficiencies in applications, ranging from electrochemical reactions to single-walled carbon nanotube (SWCNT) synthesis. While catalyst performance is highly dependent on chemistry, size, and crystal structure, their unique contributions are often hard to assess. Typical substrate-based catalyst synthesis techniques inherently couple catalyst size, composition, and concentration with substrate chemistry and conditions, as well as solvents and reactor parameters. The production of substrate-grown SWCNTs is an exemplar of this complex coupling, whereby the widely used chemical vapor deposition (CVD) requires precise control of combined substrate and catalyst conditions for effective growth. However, precise control of a specified structure—the chirality—remains exceedingly difficult. While chirality control is vital for applications in next-generation electronics (*1*) and optoelectronics (*2*), further fundamental work is required to achieve catalysts that can effectively grow chiral-specific CNTs.

For the CVD process, because of the diameter correlation between the SWCNT and catalyst (*3*, *4*), a method to precisely control catalyst sizes smaller than 2 nm is a long-sought goal. Small catalysts (≤2 nm) allow preferential growth of SWCNTs with diameters of 0.7 to 1.5 nm and further control their chirality. However, synthesis of monodisperse catalysts is currently still restricted by the dependence on substrate composition and morphology, usage of specific organic molecules and solvents, and the precise growth conditions.

Within the past few years, catalysts that remain in a solid/rigid state during CNT growth (typically 700° to 1000°C), such as W_6_Co_7_ (*5*), WC, Mo_2_C (*6*), or even Co*_x_*Mo*_y_* (*7*), have introduced new hope that in situ chirality control is possible. Contradictory theoretical discoveries attempted to explain the dominant mechanism offering chirality enrichment. A unique crystal structure (*5*, *6*, *8*) or a rather generic, universal interface (*9*–*11*) has been hypothesized, with limited evidence. To facilitate thorough understanding of the precise chirality selection mechanism, a general method to produce solid catalysts having a variety of crystal structures and compositions is therefore indispensable.

Here, we present the first study of a continuous production method of size-selected solid catalysts for SWCNT chirality control using straightforward gas-phase synthesis and selection techniques, to extend the applications of floating catalyst CVD (FCCVD) that is currently based on liquid catalysts. The monodisperse solid nanocatalysts enable SWCNT synthesis with concentrated chiral angles within 19° ± 5°. These CNT abundance results are compared with existing theories. While our findings are incongruous with the symmetry-matching theory (*5*, *6*, *8*), our results support the increasingly well-developed interface thermodynamics (*9*–*11*) and kinetic growth mechanisms (*10*). We are able to further this theory by inclusion of chiral-dependent growth and catalyst saturation parameters. These new advances are important to develop future continuous synthesis methods for chirality-controlled SWCNTs.

## RESULTS

### NP synthesis for solid catalysts

**Fig. 1:**
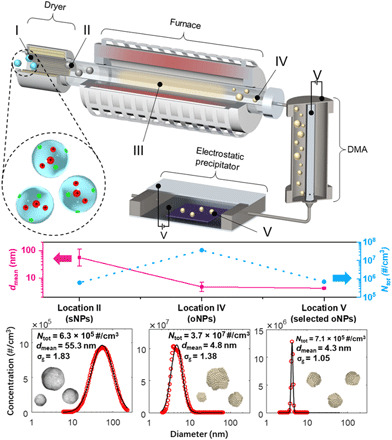
Experimental setup for continuous catalyst NP generation, size selection, and collection. Location I represents atomized solution droplets as they enter the dryer, location II shows salt NPs (sNPs) after drying, location III shows metal oxide gas after calcination and vaporization in furnace, location IV shows oxide NPs (oNPs) after nucleation, and location V shows oNPs depositing onto substrate after size selection using a differential mobility analyzer (DMA). Example evolution of geometric mean particle diameter (*d*_mean_) and concentration (*N*_tot_) of rhenium (Re) particles is shown in the middle for NPs (sNPs, oNPs, and size-selected oNPs) at locations II, IV, and V. Measurements from a scanning mobility particle sizer spectrometer are shown at the bottom with *N*_tot_, *d*_mean_, and geometric SDs (σ_g_).

Here, our solid catalyst synthesis process begins with the generation of gas-phase NP oxides of refractory metals suspended in a carrier gas, as shown in Fig. 1 (details on gas-phase synthesis and characterization can be found in section S1). These high–vapor pressure oxides allow gas-phase transport and controlled synthesis of metal oxide NPs (oNPs). Upon reduction, the refractory metal elements exhibit extremely high melting points as pure metals or carbides, allowing them to remain rigid and crystalline during CNT growth. Refractory metal catalysts within this study are focused on tungsten (W), molybdenum (Mo), or rhenium (Re) catalysts, because all of them have been demonstrated as efficient elements for CNT growth, particularly Re (*12*). This work also represents the first use of Re for chirality-controlled CNT growth. Gas-phase size distributions of all NPs are first analyzed using a scanning mobility particle spectrometer (SMPS).

An atomized salt solution (location I in Fig. 1) passes through a desiccant dryer where the water is removed. The precipitated salt NPs (sNPs; location II) form with a large mean diameter (*d*_mean_) and a wide distribution of sizes. The gas-phase sNP passes through a tube furnace, in which the sNPs decompose and are calcined to metal oxides. Because the metal oxides are supplied at flow rates below saturation, these metal oxides completely vaporize (location III) and subsequently nucleate into metal oNPs (location IV). Critically, the vaporization-and-nucleation process produces oNPs with an order of magnitude smaller size and which are nearly two orders more numerous than the initial sNPs. Their dominant sizes are also adjustable from 3 to 20 nm by varying the concentration of the atomized solution (fig. S2).

Compared with sNPs, the distribution of oNPs (location IV) is also narrower and very near to that of a “self-preserving” size distribution. However, the geometric SD (σ_g_ = 1.2 to 1.4) of oNPs is still too large [corresponding full-width half-maximum (FWHM) = 3.7 nm]. To precisely control the catalyst diameter, NP mobility selection is used to transform the distribution from polydisperse to monodisperse.

### NP size selection

**Fig. 2:**
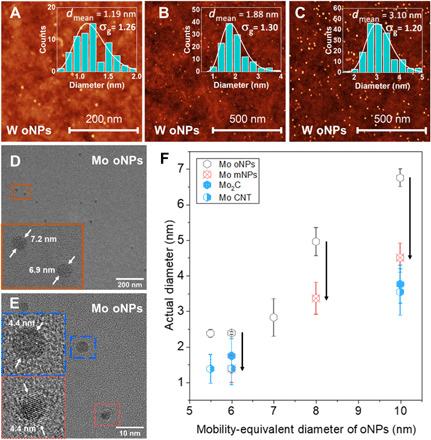
NPs after size selection and their size evolution during CNT growth. (**A** to **C**) AFM images of W oNPs homogenously deposited on SiO_2_/Si substrates. Particle populations having narrow size distributions with various mean diameters are shown including (A) ~1.2 nm, (B) ~1.9 nm, and (C) ~3.1 nm. Also shown are high-resolution transmission electron microscope (HRTEM) images of (**D**) as-produced polycrystalline Mo oNPs with a mobility-equivalent diameter of 10 nm corresponding to ~7 nm diameter measured by TEM and AFM, and (**E**) corresponding single-crystal Mo mNPs after reduction and reconstruction. mNP diameter is ~60% of the former oNP diameter. The plot in (**F**) presents the evolution in observed Mo NP diameter through various particle production steps and CNT growth for several oNP mobility-equivalent diameters, as prescribed using the DMA. The black arrows demonstrate the ~60% diameter ratio between (D) and (E). During the growth process, the resultant catalysts (here, Mo_2_C) retained the size of mNPs and strongly correlate to their nascent CNTs’ diameters.

This work extends from our own (*13*, *14*) use of an SMPS as a characterization tool [similar to studies from other groups (*15*, *16*)] to analyze gas-phase particles from a sample flow. In this work, we use the differential mobility analyzer (DMA) as an integrated component within the process for the entire aerosol flow, which allows the proactive selection of NPs by diameter for further use. In this process, after being charged by a radioactive neutralizer, the oNPs are passed between two concentric cylinders in the DMA at an electrical potential difference. Only oNPs with the prescribed mobility-equivalent diameter pass downstream, which results in the narrow size distribution of selected oNPs (more details are provided in section S1). Using atomic force microscopy (AFM), the size distribution of these selected oNPs is measured (Fig. 2, A to C) to be precisely tuned in the range of 1 to 3 nm. Reduction of the oxidation state after size selection allows for further reduction in catalyst size while retaining the narrow distribution (Fig. 2F).

The distribution after selection is narrow with a σ_g_ = 1.05 (σ_g_ = 1 represents ideal monodisperse particles). Moreover, the FWHM of these selected distributions scales with the selected midpoint size. Therefore, selection of smaller particles also corresponds to a smaller FWHM, which is the key feature for achieving nearly monodisperse catalysts for SWCNT growth from a continuous process. On the basis of the flow conditions used here, σ_g_ = 1.05 means that the FWHM of the size distribution is 7.5% of the peak size. These narrow distributions are also verified by high-resolution transmission electron microscope (HRTEM; Fig. 2, D to F).

### NP evolution during CNT growth and effects of catalyst diameter constraint

The monodisperse oNPs are collected by an electrostatic precipitator (location V) onto various target substrates with a uniform and tunable areal density. At the point of collection, the NPs are composed of oxidized metals (more composition characterization can be found in fig. S3), which from HRTEM (Fig. 2D, inset) appear to be closely packed polycrystals. After reduction at high temperature in H_2_, the oNPs are reconstructed into single-crystal solid metal NPs (mNPs; Fig. 2E). In Fig. 2F, with Mo as an example, the HRTEM results of the catalyst size distributions at various process stages are summarized. The reduction and reconstruction reduce the mNP diameter to ~60% of the diameter of as-collected oNPs (black arrows in Fig. 2F).

**Fig. 3:**
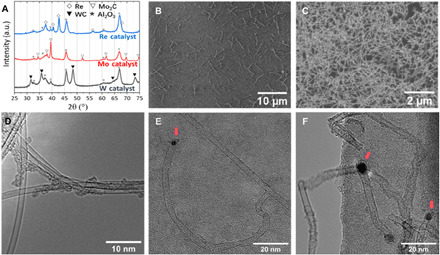
The constraint effect of catalysts on the areal density and diameter of CNTs. (**A**) XRD profile of real W, Mo, and Re catalysts during the growth process; all NPs are supported on alumina filters; SEM images of SWCNTs grown from low (**B**) and high (**C**) areal density of NPs on marked SiO_2_/Si substrate; with the small size of catalysts, i.e., (**D**) 1.4-nm Mo catalysts and (**E**) 2.6-nm W catalyst, high-quality SWCNTs are produced. Larger oNPs, i.e., (**F**) 4.5-nm Mo catalysts, produced few-walled CNTs with more defects. Tangential growth is the dominant mechanism. Catalysts are all observed to be wrapped by a carbon layer, forming a “pea pod” morphology, indicated by arrows in (E) and (F). With a size of less than 2 nm, catalysts are too small to be distinguished from the grid background, as happened in (D). a.u., arbitrary units.

Upon introduction of the carbon feedstock, solid catalysts are converted to their final forms, which induce CNT growth. X-ray diffraction (XRD) patterns presented in Fig. 3A show that, following carbon exposure, W and Mo form carbides—WC and Mo_2_C, respectively. Notably, Re-based catalysts behave uniquely by retaining their elemental metal state (more details in fig. S3). WC, Mo_2_C, and Re all have very high melting points (for bulk material *T*_WC_ = 2785°C, *T*_Mo_2_C_ = 2687°C, and *T*_Re_ = 3186°C), which guarantee their solid state during growth. Moreover, Mo_2_C is an orthorhombic crystal with the space group of *Pbcn* (point group *D*_2*h*_). By contrast, WC and Re are hexagonal crystals with the space group of *P*6*m*2 (point group *D*_3*h*_) and *P*6_3_/*mmc* (point group *D*_6*h*_), respectively. The difference of crystal structure and particularly symmetry presents a unique opportunity to investigate possible mechanisms of chirality control with solid catalysts under similar size and CNT growth conditions.

Because of the gas-phase nature of the NP formation process, by modifying the NP collection time, low and high areal densities of resultant SWCNTs can be simply achieved (Fig. 3, B and C). This also allows for straightforward deposition and growth on various substrates without any ex situ transfer processes (fig. S4). By decoupling the catalyst size from their areal density, further growth of high-density SWNT arrays for electronics becomes possible (*17*, *18*).

On the basis of the summary of HRTEM results (Fig. 2F), the diameters of catalysts are consistent with the corresponding mNPs, as are the diameter of the grown CNTs. With ~4.5-nm catalysts, few-walled CNTs with numerous kinks are the dominant product with lengths of tens of micrometers (Fig. 3F). With ~2.6-nm catalysts, longer and higher-quality SWCNTs with diameter ~3 nm are obtained (Fig. 3E). CNTs show tangential growth from the solid catalysts (Fig. 3, E and F), while the catalyst maintains its original diameter and spherical shape. It does not undergo the common “reshaping” process frequently seen for liquid catalysts (*19*, *20*). With ~1.4-nm catalysts, SWCNTs with diameter ~1.5 nm dominate the product (Fig. 3D). These results reconfirm the dominant role of catalyst size on the resultant SWCNT diameter (*21*) and ultimately by the size of oNPs controlled by our process. These small and narrowly distributed solid catalysts (normally <1.5 nm) from our process and their different crystal structures also limit the range of accessible chiralities for SWCNTs.

### FCCVD growth of CNT using solid catalysts

**Fig. 4:**
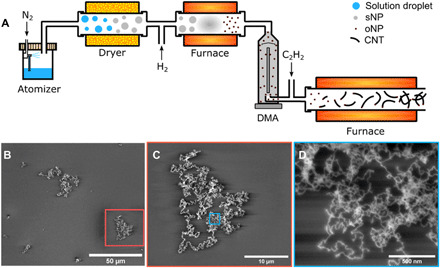
FCCVD growth of CNT using solid catalysts. (**A**) Schematic diagram of FCCVD setup and (**B** to **D**) the corresponding preliminary results of CNTs grown from FCCVD without size selection.

As mentioned above, realization of the continuous production of chirality-controlled SWCNTs is a long-sought goal. Because of the gas-phase nature of our NP formation process, the catalyst preparation is both continuous and substrate independent (*6*). Considering the advanced FCCVD production of CNTs using more traditional catalysts such as iron, our method can be directly integrated into this style of process to achieve the first documented fully continuous production of CNTs using solid catalysts (Fig. 4A).

By introducing hydrogen in-line, an aerosol of oNPs would be directly reduced to an aerosol of mNPs. With the addition of a carbon feedstock (C_2_H_2_), real catalysts of final composition would form and support the growth of CNTs in a suspending state in the carrier gas. In Fig. 4 (B to D), CNTs produced via the described path using WC catalysts are shown. Currently, the CNTs from FCCVD are still dominated by few-walled CNTs with numerous kinks. The primary reason for the production of few-walled CNTs is the relatively large catalysts currently used compared with those used for substrate growth. The larger particles allow for higher concentrations, offsetting the much lower concentration of smaller catalysts after multiple losses during the FCCVD process (further details in section S1). Further refinement should reduce losses to realize the FCCVD growth of chirality-controlled SWCNTs with solid catalysts.

### Chirality distribution from solid catalysts

**Fig. 5:**
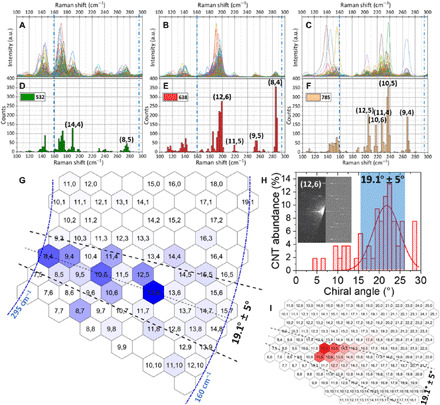
Chirality distribution of SWCNTs grown from solid catalysts. Raman mapping results of SWCNTs grown from WC catalyst (diameter, ~1.5 nm). Raman spectra in the RBM region detected by (**A**) 532 nm, (**B**) 638 nm, and (**C**) 785 nm with all baselines subtracted. (**D** to **F**) The peak position abundance statistics results from three lasers are summarized with normalized scale. After converting peak position abundance to chirality abundance based on the Kataura plot (fig. S1), the results are displayed on a map (**G**). To guarantee unambiguous identification, the painting is done only for SWCNTs with 0.81 nm < diameter < 1.53 nm [160 cm^−1^ < RBM peak <295 cm^−1^, marked by the blue dash-dotted line in (A) to (G)]. Chirality cells that are not resonant with three lasers are left empty in (G). Chiralities near (2*n*,*n*) are most enriched (corresponding range of 19.1° ± 5° is marked by a black dashed line), and few can be found near the zigzag region. (**H**) The chiral angle statistics measured with nanobeam electron diffraction (ED) with the most dominant being in the same 19.1° ± 5° range; (inset) the example ED pattern of the (12,6) tube is shown with its simulation pattern (left, experiment; right, simulation). (**I**) The qualitative calculation of abundance based on interface thermodynamics, kinetic growth theory, and the extended chirality-dependent growth time determined from this work. All factors, particularly the chirality-dependent growth time theory (detailed in section S4), lead to further concentrated abundance around (2*n*,*n*).

Resonant Raman spectroscopy shows the RBM (radial breathing mode) peaks of SWCNTs in the laser spot excited with laser phonons (*22*, *23*), facilitating the chirality identification of SWCNTs. Here, with Raman *x* − *y* two-dimensional mapping results from 532-, 638-, and 785-nm lasers, respectively (Fig. 5, A to C; experiment details are presented in section S2), the resonant peak position abundance statistics results are summarized with a normalized scale (Fig. 5, D to F). After converting peak position abundance to chirality abundance based on a rectified Kataura plot (*24*, *25*) (fig. S1), the chirality abundance results are displayed in the map (Fig. 5G).

With size-selected WC (*d* ≈ 1.5 nm) catalysts as an example, it is apparent from Fig. 5G that the most enriched visible chiralities are (12,6), (10,5), (8,4), (12,5), and (9,4), which are all on or near (2*n*,*n*), with a chiral angle near 19.1°. The highest abundance of (12,6), (10,5), and (8,4) reached approximately 39, 23, and 12%, respectively, among the diameter range of 0.81 to 1.53 nm (160 cm^−1^ < RBM peak < 295 cm^−1^). All visible (2*n*,*n*), (2*n ±* 1/2*n*,*n*), and (2*n ±* 2/2*n ±* 1/2*n*,*n*) chiralities reached combined abundances of ~74, ~81, and ~90%, respectively. Chiralities with a chiral angle within 19° ± 5° reached an abundance of 93.7% within small-diameter tubes. In contrast, few CNTs have chiralities near the zigzag or armchair regions.

The chirality enrichment around (2*n*,*n*) can also be seen from chiral angle statistics measured with nanobeam electron diffraction (ED) (*26*) in Fig. 5H. Tubes with a chiral angle of 19° ± 5° are still enriched. Some tubes with small chiral angle (near zigzag) appeared, while zero armchair tubes (chiral angle = 30°) are identified. Results from ED slightly differ from the identification from Raman mapping since the results are not based on total abundance. Tubes that protrude from or stretch across the substrate are examined with ED, and therefore, abundance weighting on tube length is reduced while more insight into tube numbers can be gained. We attribute this appearance of near-zigzag tubes to the enrichment during nucleation (*11*). The slightly broader chirality distribution from ED compared with Raman mapping is indicative of the broad CNT nucleation probability distribution.

Compared with WC catalysts, Mo_2_C and Re not only show similar trends and produce more near-(2*n*,*n*) chiralities (fig. S6, E to L) but also exhibit distinct chiral preference. Regardless of the catalyst compound and its associated crystal structure, the highest chirality abundances are those around (2*n*,*n*), while near-zigzag and near-armchair chiralities are the rarest (Fig. 5 and fig. S6). (12,6) and (10,5) SWCNTs with their respective sixfold and fivefold symmetries both show considerable abundance despite their symmetry mismatch with their host catalysts.

In addition, the carbon-to-hydrogen ratio (C:H) in the CVD gas composition influences the chirality distribution. Only with excess carbon supply (C:H > 1:15) is the enrichment around the (2*n*,*n*) line apparent. In contrast, when C/H < 1:5 (fig. S7), chirality control is less obvious. In this case, many other chiralities are observed in an abundance comparable to those near the (2*n*,*n*) line.

## DISCUSSION

### Chirality-dependent growth life on solid catalysts

The mechanisms underlying the chirality selection of SWCNTs are of great interest to the scientific community with a recent proliferation of theoretical and numerical contributions describing varying aspects of chirality selection and growth. Newly developed solid catalysts particularly require further investigations to determine the dominant mechanism of selective growth, given their outstanding performance. Recent useful contributions include (i) interface energy–driven nucleation thermodynamics (*9*–*11*), (ii) interface configurational entropy–driven nucleation thermodynamics (*27*), (iii) cap nucleation kinetics with Zeldovich factor (*28*), (iv) catalyst epitaxy with symmetry matching (*5*, *6*, *8*), (v) CNT growth kinetics (*10*), and (vi) etching agent–dependent growth kinetics (*29*), among others. These contributions provide a useful set of potential theoretical mechanisms and physical parameters that must be measured and tested to refine the varied contributions into a cohesive and useful theory that can guide the development of models, experiments, and production of chirality-controlled CNTs.

The production and precise size control of solid catalysts for CNT growth provide a means to examine and refine theories regarding the abundance of CNT chiralities by limiting multivariant factors such as variable chemistry, interface energy, and catalyst size. By limiting the degree of freedom, investigations can also focus on variations in chirality led by growth environment and time differences.

Our present investigations allow us to examine several existing mechanisms. It is apparent that the ability for a catalyst NP to remain solid and to maintain its shape/diameter during growth is the key feature for chirality enrichment around the (2*n*,*n*) line. This trend has been observed for three different catalyst compositions having different crystal symmetries (point groups). Catalyst symmetry (iv) seems to have no discernible influence on the CNT chirality abundance. Previous reports (*9*–*11*, *27*) argue that tight matching between tubes and catalysts would constrain the growth rate of tubes and may limit instead of enriching the abundance.

The enriched chiralities near (2*n*,*n*) support the CNT growth kinetics mechanism (v), but with an even stronger trend when comparing fig. S9E with Fig. 5G. Comparing the ED results with those from Raman mapping, the appearance of near-zigzag tubes shows general agreement with interface energy–driven nucleation thermodynamics (i) despite lacking precise inputs for critical values, such as catalyst interface energy (fig. S9D). The slightly broader chirality distribution from ED compared with Raman mapping follows the recently reported A|Z segregation on the interface of solid catalyst tubes, which broadens the CNT nucleation probability distribution. By comparing the results from three different catalysts, the subtle variations can likely be attributed to differences in tube-catalyst interface energy and the consequently different nucleation (i) and growth of CNTs (v).

To explain the additional enrichment of near-(2*n*,*n*) chiralities compared with the prediction based on growth kinetics mechanism, we extend the theory (*9*–*11*) with chirality-dependent growth time (Fig. 5I; additional details are in section S4.). In addition to being preferred by kinetics, these near-(2*n*,*n*) chiralities also prolong their growth time before being halted from catalyst poisoning by an abundance of carbon (catalysts frequently found to be encapsulated by a layer of carbon; Fig. 3, E and F, and fig. S8 indicated by arrows). Thus, the length of a CNT would not only be constrained by growth speed but also be determined by the chirality-dependent growth time. In addition, the size of catalyst determines the region of possible chiralities, thus affecting final chirality abundance.

Although the inclusion of catalyst poisoning within existing theoretical framework improves theoretical prediction of results concurrent with our experiments, the current theories are still not robust in explaining all observed phenomena. More experimental studies—particularly the in situ TEM observation of single tube growth based on size-selected solid catalysts—are needed to further test and refine existing models, e.g., time-dependent growth. Our results show a need for inclusion of catalyst deactivation as a parameter that influences CNT chiral abundance. Also, we demonstrate that currently unmeasured or simulated constants, such as interface energy, must be examined to advance our understanding of the chirality selection and growth process.

To date, methods to produce solid catalysts are batch processes that rely on either specialized, expensive precursors or specific substrate compositions. Gas-phase synthesis of solid or liquid catalysts allows for continuous production of physically selected NPs that can be directly deposited onto any substrate material. The resultant high throughput also increases the application potential for high-throughput screening, automated catalyst design, etc.

The solid state of the reported catalyst during reduction and growth steps suppresses Ostwald ripening and contributes to a reliable and predictable catalyst size as selected. In contrast, because of Ostwald ripening and flexible morphology of traditional liquid catalysts, chiralities grown are inherently diverse.

Beyond the three metals studied here, many others may also be viable candidates for solid catalysts, including other refractory metals such as V, Nb, Ru, Rh, and Os. The extensive list of compositions can offer different interface energies at the catalyst-tube interface, providing the enrichment of other chiralities in the final products with our compatible experimental setup.

Extension of these techniques can achieve further reduced catalyst size (<1 nm) and narrower size distributions, which would further prohibit perpendicular growth of SWCNTs. Combined with the difference in chirality-dependent growth times, semiconducting chiralities such as (8,4), (9,4), (10,3), and (11,3) are predicted to dominate the final products (fig. S9i) to achieve high-purity semiconducting SWCNTs product with a simple direct growth method.

## CONCLUSIONS

This work presents the first documented continuous production of solid catalysts for SWCNT chirality control using an aerosol-based size selection method. This is accomplished through synthesis of the oNPs of W, Mo, and Re. During CNT growth, these NPs are reduced, forming the solid catalyst and can produce well-controlled CNT diameter and chirality distributions. This work also marks the first chirality-controlled growth of SWCNTs from Re. We attribute our chirality control to the differences in growth rate between chiralities, further amplified, as we show, by chirality-dependent growth time. With a high carbon growth environment, all three catalysts with different symmetry generate similar near-(2*n*,*n*) chirality SWCNTs, convincingly ruling out the symmetry-matching theory. All visible (2*n*,*n*), (2*n ±* 1/2*n*,*n*), and (2*n ±* 2/2*n ±* 1/2*n*,*n*) chiralities reach a combined abundance of ~74, ~81, and ~90%, respectively. We report the first growth of CNTs from an FCCVD process using a solid catalyst. With this aerosol method for NP production, continuous synthesis of chirality-controlled SWCNTs can be achievable and may help increase the impact of this remarkable material in many fields of science and engineering.

## MATERIALS AND METHODS

Materials and methods details are available in sections S1 and S2.

### NP synthesis, size selection, and collection

Aqueous metal salt solutions are prepared for (NH_4_)_6_H_2_W_12_O_40_ (Sigma-Aldrich, 463922), (NH_4_)_6_Mo_7_O_24_ (Sigma-Aldrich, 09878), or NH_4_ReO_4_ (Sigma-Aldrich, 316954). The solution is atomized into droplets suspended in nitrogen carrier gas through an atomizer (TSI Inc. 9302) and dried in a desiccant dryer, leaving precipitated sNPs. The sNPs are then sent through an alumina tube in a furnace whose temperature is set to 950°C; the nucleated polydisperse oNPs are first charged using a radioactive charge neutralizer (TSI 3077) and then sent through a DMA (TSI 3085). The DMA selects particles with a prescribed mobility-equivalent diameter and a very narrow range. The DMA-selected NPs are sent into an electrostatic precipitator for collection onto the target substrate.

### CNT growth on substrate and FCCVD growth

Substrate-based CVD growth of SWCNTs begins by reducing oNPs in H_2_ with temperature slowly rising to 450°C to obtain mNPs. Ethanol vapor as the carbon feedstock is introduced into the reaction zone (850°C) in an Ar carrier gas. After a set growth time, the carbon-rich environment is expelled by H_2_, and the samples are cooled to room temperature. For FCCVD growth, oNP aerosol (later with reaction gas) is sucked into the furnace by a pump after the furnace exit. H_2_ is then introduced in-line, and oNP aerosol would be directly reduced to an aerosol of mNPs. With the addition of a carbon feedstock (C_2_H_2_), real catalysts would form and support the growth of CNTs while still suspended in the carrier gas. Grown CNTs are collected at the downstream of the growth furnace onto SiO_2_/Si substrates using thermophoretic deposition.

### Aerosol characterization, AFM, HRTEM, ED, XRD, and Raman mapping

Aerosol size distributions of all NPs are analyzed using an SMPS, which consists of a combination of a DMA (TSI 3085) and a condensation particle counter (TSI 3756). AFM is conducted on a Veeco Dimension Pro AFM on Peakforce mode. HRTEM is conducted on an FEI Talos F200X TEM (200 kV for NPs and 80 kV for CNTs) with oNPs collected onto Si_3_N_4_ grids. The reduction and CNT growth processes are also conducted in situ on the Si_3_N_4_ grids before characterization. ED of suspended CNTs is conducted on FEI Tecnai F20 FEG and Talos F200X TEMs and working on 80 kV with scanning TEM nanobeam mode. For XRD characterization, the oNP aerosols are vacuum-filtered onto Anodisc aluminum oxide membrane filters (AAO; Whatman FIL3010). To obtain the NP composition at different steps during the growth process, the oNPs on AAO are reduced and later used for typical CNT growth. Raman mapping is conducted in the RBM range, with 532-, 638-, and 785-nm lasers. The step size is set to 3 μm in both the *x* and *y* directions.

## Supplementary Material

abb6010_SM.pdf

## SUPPLEMENTARY MATERIALS

Supplementary material for this article is available at http://advances.sciencemag.org/cgi/content/full/6/40/eabb6010/DC1
