# Supplementary material for: High-precision solid catalysts for investigation of carbon nanotube synthesis and structure
Source: Sci Adv. 2020 Sep 30;6(40):eabb6010. doi: 10.1126/sciadv.abb6010 (PMC7527216; doi:10.1126/sciadv.abb6010)
Supplement: abb6010_SM.pdf [file abb6010_SM.pdf]

Science Advances
AAAS

advances.sciencemag.org/cgi/content/full/6/40/eabb6010/DC1

Supplementary Materials for

High-precision solid catalysts for investigation of carbon nanotube synthesis and structure

Xiao Zhang, Brian Graves, Michael De Volder*, Wenming Yang, Tyler Johnson, Bo Wen, Wei Su, Robert Nishida, Sishen Xie, Adam Boies*

*Corresponding author. Email: amb233@cam.ac.uk (A.B.); mfld2@cam.ac.uk (M.D.V.)

Published 30 September 2020, *Sci. Adv.* **6**, eabb6010 (2020)
DOI: 10.1126/sciadv.abb6010

This PDF file includes:

Sections S1 to S4
Table S1
Figs. S1 to S9
References

S1 Method details - Aerosol technology on particle generation, sieving, characterization and CNT growth on substrate

Aerosol Characterization

Aerosol size distributions of all NPs are first analysed using a Scanning Mobility Particle Spectrometer (SMPS), which consists of a combination of a Differential Mobility Analyzer (DMA, TSI 3085) and a Condensation Particle Counter (CPC, TSI 3756). The DMA scans through its particle size range and the CPC records the corresponding number concentration of each size bin, specifically the particles' mobility-equivalent diameter. The vertical axis $dN/d\log d_m$ represents the number concentration of that bin normalized by bin width. Measured data is fitted using lognormal distributions as is standard practise in aerosol science, giving the Geometric Mean Diameter (GMD, peak position, d_{mean} in main text), Geometric Standard Deviation (GSD, width, σ_g in main text) and total number concentration (N_{tot}, area under distribution) of the sampled aerosol.

Nanoparticle Synthesis

Highly dilute aqueous metal salt solutions are prepared for $(NH_4)_6H_2W_{12}O_{40}$ (Sigma-Aldrich 463922), $(NH_4)_6Mo_7O_{24}$ (Sigma-Aldrich 09878), or NH_4ReO_4 (Sigma-Aldrich 316954) with concentrations of 0.3 mM with respect to the metal atoms. The solution is atomized into droplets suspended in nitrogen carrier gas using a flow rate of 3.6 slpm through an atomizer (TSI Inc. 9302). The resultant mist contains solution droplets that have a diameter of approximately 1.5 µm. The mist then passes through a

desiccant dryer to remove the water, leaving precipitated salt nanoparticles (sNPs) suspended in the nitrogen. The example in Figure 1 shows Re sNPs with d_{mean} = 55 nm and σ_g = 1.83. This distribution is very wide considering that at equilibrium, lognormally distributed aerosol size distributions should possess σ_g = 1.4.

The sNPs are then sent through an alumina tube in a furnace whose temperature is set to 950°C, in which salt molecules decompose and being calcined to form metal oxides. The metal oxides then completely vaporize in the hottest zone of the furnace and then nucleate into oNPs upon cooling. With the vaporization and subsequent nucleation process, the oNPs are an order of magnitude smaller in size (in the example, the d_{mean} = 4.8 nm), and nearly two orders more numerous than the preceding sNPs. The distribution is also narrower with σ_g = 1.38, very near to that of an equilibrium or "self-preserving" size distribution. Given this d_{mean} and σ_g, the full-width half-maximum (FWHM) of the distribution is 3.7 nm.

Using the very diluted salt solution, rather than heat elemental metal, we realize facile and accurate control on the input amount of NP precursors, and the precise control on the size at location IV in Fig. 1, main text.

Nanoparticle Size Selection

To produce a nearly-monodisperse population of particles for collection, the polydisperse oNPs from the furnace are first charged using a radioactive charge neutralizer (TSI 3077) and then sent through a DMA (TSI 3085). The nanoparticles move axially through the annular region between the DMA cylinders with the carrier gas but are also pulled radially inwards by the electric field. The DMA selects particles with a prescribed charge to drag ratio, known as mobility-equivalent diameter. The drag against the particles' radial velocity dictates how quickly the particles migrate radially inwards. The balance between the drag force and electric field force results in a very narrow range of particles based on their charge to drag (diameter) ratio. In the example, the DMA output distribution at location V is set to a prescribed d_{mean} = 4.15 nm. SMPS scans show excellent agreement to this setting since the resultant d_{mean} = 4.31 nm. The distribution is extremely narrow (nearly monodisperse) with σ_g = 1.05. Note that σ_g = 1 represents an infinitely thin distribution of particles at exactly one size (perfectly monodisperse). With σ_g = 1.05, FWHM of the size distribution should be 7.5% of the peak size. For the setpoint of 4.15 nm this corresponds to an extremely narrow FWHM of 0.31 nm. In reality the distribution broadens slightly and is approximately 0.52 nm for the above example. Uniquely, this method allows selecting a monodisperse population of particles in a wide range of sizes.

During the DMA size selection, the aerosol flow rate is set to 1.5 lpm, and the DMA's sheath flow is set to its maximum of 20 lpm to produce the narrowest particle size range (the DMA's transfer function is explained in more detail in 0).

Nanoparticle Collection

To collect the nanoparticles for substrate-based growth or characterization, the DMA-selected nanoparticles (which remain charged) are sent into an electrostatic precipitator (location V), in which the trajectory of nanoparticles are deflected by the electric field (5-300 V/mm) and deposited onto the target substrate. The concentrations of nanoparticles before and after precipitator are monitored by a CPC to roughly estimate deposition gains in the precipitator. The homogenous electric field collected the NPs onto various target substrates with a uniform areal density (verified by AFM results).

The areal density of oNPs (n) on the substrate could easily be tuned by changing the deposition time (t), considering aerosol concentration (N), collection efficiency (η), aerosol volumetric flow (V) and size of substrate (A): $n = NVt\eta/A$.

CNT Growth on Substrate

Substrate-based CVD growth of SWCNTs begins by reducing various substrate-deposited oNPs in H_2 through a temperature programmed reduction processes (slowly rising to 450°C in H_2) to obtain solid metal-nanoparticles (mNPs) catalysts. Ethanol vapour as the carbon feedstock is introduced into the reaction zone (850°C) in an Ar carrier gas. The H_2 flow rate is varied to tune the carbon to hydrogen ratio (C : H) of the growth environment. After a set growth time (normally 20 min), the carbon-rich environment is expelled by H_2, and the samples are cooled to room temperature. The growth time is set to be longer than 10 min to guarantee a steady-state production result.

FCCVD Growth of CNT Using Solid Catalyst

After verifying steady production of oNPs, (same parameters as shown in the above “Nanoparticles Synthesis” section), 0.2-1 lpm of aerosol (later with reaction gas) is sucked into the furnace for CNT growth by a pump after the furnace exit. Hydrogen (1-2 lpm) is then introduced in-line, and oNPs aerosol would be directly reduced to an aerosol of mNPs. With the addition of a carbon feedstock (C_2H_2, 5-50 sccm), real catalysts would form and support the growth of CNTs while still suspended in the carrier gas. Grown CNTs are collected at the downstream of the growth furnace onto SiO_2/Si substrates using thermophoretic deposition.

As a preliminary result using novel solid catalysts to grow CNTs by floating catalyst CVD (FC-CVD), the current production of multiwalled tubes is mainly caused by the relatively larger catalysts used (4-6 nm) compared with those used for substrate growth (mainly 1-2 nm). This was done because of the compromise between catalyst size and concentration for floating catalyst growth at the current stage. The sources of loss during FCCVD for catalysts (especially for size range of 1-3 nm) include: (i) much smaller proportion of oNPs being charged by the neutralizer as the size becomes smaller (only charged oNPs can be size selected and collected); (ii) discarding catalysts with other size during size selection; and (iii) diffusion loss and electrostatic loss in the growth chamber. Production suffering from all the losses simultaneously will result in too few SWCNTs to collect and characterize in any reasonable timeframe. Further dedicated work is in progress to reduce losses and to finally realize the floating growth of chirality-controlled SWCNTs with solid catalysts.

S2 Method details - AFM, HRTEM, ED, XRD, and Raman Mapping

AFM is conducted on a Veeco Dimension Pro AFM on Peakforce mode.

HRTEM is conducted on FEI Talos F200X TEM (200 kV for NPs, 80 kV for CNTs) with oNPs collected onto Si_3N_4 grids. The reduction and CNT growth processes are also conducted in-situ on the Si_3N_4 grids before characterization.

Electron diffraction (ED) of suspended CNTs is conducted on FEI Tecnai F20 FEG and Talos F200X TEMs and working on 80 kV with STEM nanobeam mode. The Si_3N_4 grid with CNTs grown is punctured on purpose, to obtain suspended CNTs stretching out or suspended across the sawtooth bay of the

broken grid occasionally. Consequently, ED illustrates more information regarding tube number than tube length, thus disclosing additional clues pertaining to nucleation conditions.

For XRD characterization, the oNPs aerosol are vacuum filtered onto Anodisc aluminium oxide membrane filters (AAO, Whatman FIL3010). The oNP size is controlled by the concentration of the aqueous salt solution. To obtain the NP composition at different steps during the growth process, the oNPs on AAO are reduced, and later used for typical CNT growth. These are then used as XRD samples for mNPs and the final catalyst composition during growth.

Raman mapping on randomly distributed SWCNTs

Raman mapping is conducted in the RBM range (70-350 cm^{-1}), with 532, 638, and 785 nm lasers. Details are presented in Table S1. As an efficient characterization method for large area statistics of thousands of SWCNTs, in Raman x-y 2D mapping, the laser spot raster-scans randomly-oriented CNTs grown on SiO_2/Si substrates with location marks. The step size is set to 3 µm in both the x and y directions. With Raman xy-2D mapping, a spectrum is obtained for each pixel, which allows for a frequency of SWCNTs to be detected which is proportional to the number and length of each tube. Here, mapping results are cumulatively presented in Fig. 5a-c. The abundance corresponds to the frequency of occurrence of a corresponding peak than the RBM peak intensity (Fig. 5d-f). In other words, the abundance corresponds to the frequency of occurrence of a corresponding peak, rather than the RBM peak intensity (details presented in S2). Some chiralities show larger peak intensities due to better resonance, and this is not necessarily indicative of a higher abundance. The peak position of each spectrum in the RBM Raman map are identified after background removal (SiO_2/Si signal) and are then associated with chiralities based on the Kataura plot. Abundance statistics obtained by different lasers are normalized by pixel number and laser spot area. The statistical abundance from Raman is also summarized on the graphene map (Figure 5g, chiralities that cannot be excited by the three lasers are left with unmarked cells).

Table S1: Experimental details for Raman analysis

λ (nm)	N.A.	Power (mW)	Grating (gr/mm)	Setup
532	0.50	6.25	1800	Horiba Xplora Plus
638	0.50	7.5	1800	Horiba Xplora Plus
785	0.75	10	1200	Bruker Senterra

Rectified Kataura Plot and Abundance Analysis

It is well known that to determine the (*n,m*) based on the Kataura plot, both E_{ii} and the ω_{RBM}–d_t relation are strongly affected by the environments in which the CNTs are located. Using the Kataura plot with the proper environment rectification is vital to get reliable identification.

Here, we use the rectified Kataura plot as shown in Figure S1. For all the data, we start from the experimental data of nanotube optical transitions developed from suspended single isolated SWCNTs, based on electron diffraction and Rayleigh scattering(*25*). We modified all the E_{ii} with a redshift of 40 meV to account for the environment interaction between SWCNTs with silica substrates(*25*). The resonance window (broadening factor) is set to 100 meV(*30*). Some metallic thin SWCNTs may have a larger value because of exciton-phonon coupling(*31*), which is also considered during (*n,m*)

identification. As to the ω_{RBM}–d_t relationship, we follow the reported ω_{RBM}=235.9/d_t+5.5 for SWNTs grown on SiO_2/Si substrates(*32*).

In the Kataura plot, the chiralities with diameter > 1.53 nm (RBM peaks less than ~ 160 cm^1) are too densely located to unambiguously identify the chirality indices. Here, to guarantee unambiguous identification, graphene map painting is done only for the small diameter region, but the abundance values do account for all possible SWCNTs. Chiralities that cannot be excited by the three lasers used here are left with unmarked cells.

To account for the visible percentage of chiralities, and multiple same chirality tubes under the same laser spot, we follow the reported method(*32*) to quantify the percentage of (*n,m*) SWNTs within the range of 0.81 nm (295 cm^{-1}) –1.57 nm (160 cm^{-1}), considering the detection range of Raman and SiO_2/Si background disturbance. An average of 14.8 tubes/laser spot are observed based on SEM images.

For all visible (*2n,n*), (*2n±1,n*) and (*2n±2,n*) chiralities, each compared with tubes with similar diameter, only (10,4), (11,5) are less enriched. Considering the corresponding band gap E_{11}^{L} of (10,4) and (11,5) are located near the edge of the 0.1 eV resonance window of the 532 or 638 nm lasers, the small abundance still seems to originate from the poor resonant condition, regardless of the reported larger resonant window because of the exciton-phonon coupling(*31*). E_{11}^{H} of (11,5) also seems weakly resonant with the 532 nm laser. For chiralities outside of the (*2n,n*) region, only (8,7) and (11,10) have a moderate abundance, provided the abundances are not over-estimated by the adjacent (10,5) and (13,8)/(14,7)/(15,5) tubes, respectively.

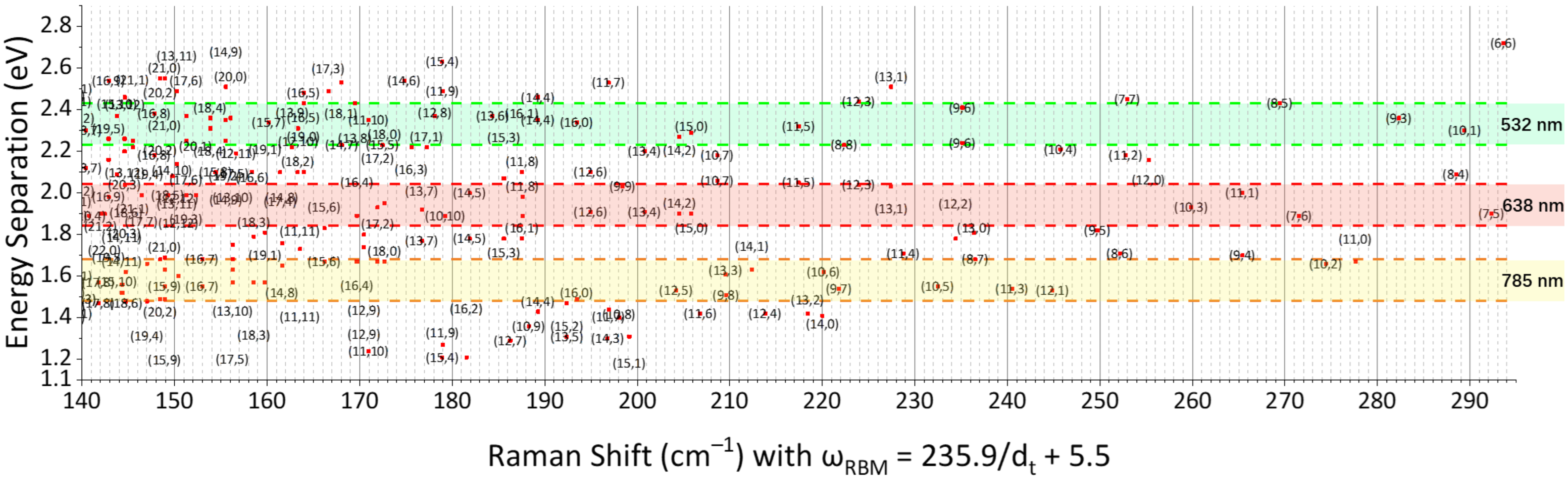

Figure S1: Part of the rectified Kataura plot for SWCNTs arrays directly grown on SiO_2/Si substrates. 532 nm, 638 nm, and 785 nm laser resonance regions are also marked with dashed lines, the broadening factor is set to 100 meV each.

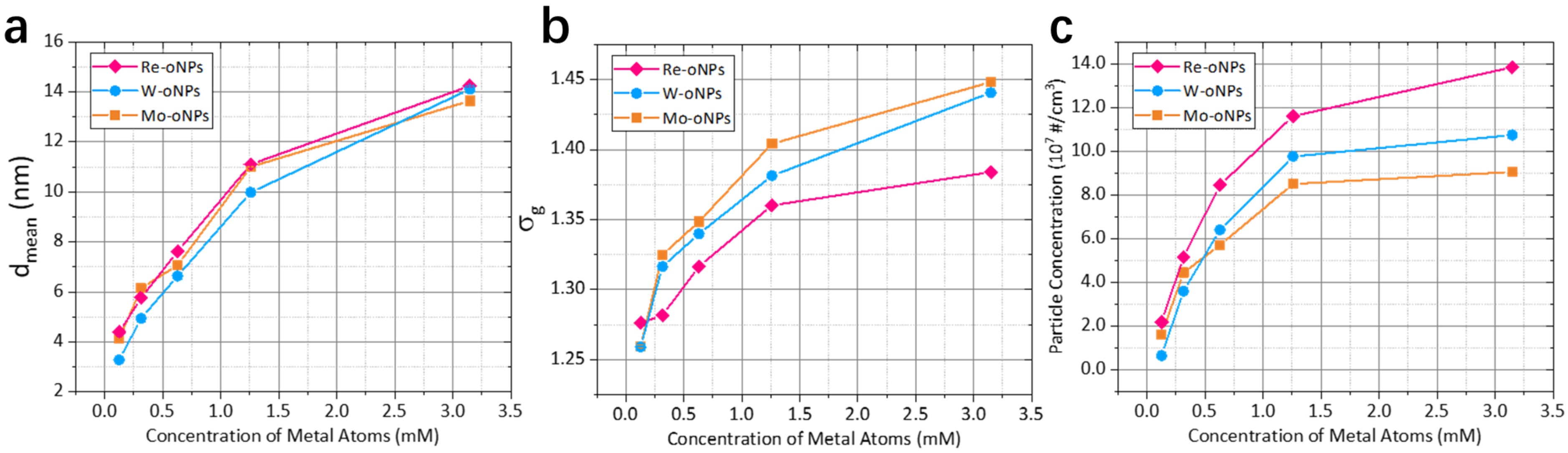

Figure S2: Rough control of oNPs before size selection by varying the concentration of the atomized solution. The relationship between concentration of atomized solutions and (a) the mean diameter (d_{mean}), (b) geometric standard deviation (σ_g), and (c) concentration of primary oNPs (measured at location IV in Fig. 1). By tuning the concentration of the atomized solution, preliminary control of the oNP size distribution can be achieved.

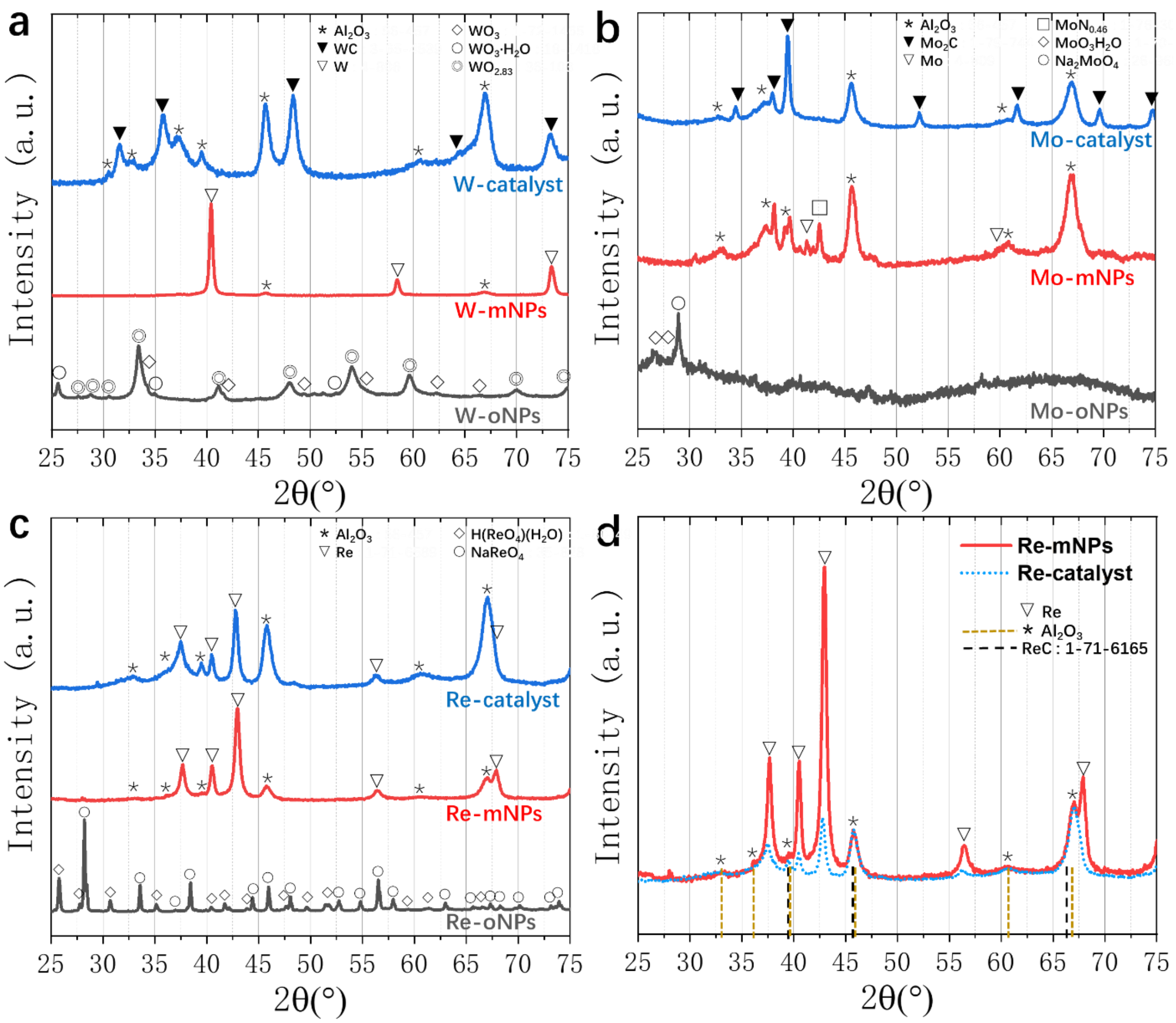

Figure S3: XRD patterns of nanoparticles/catalysts at different steps for CNT growth. (a) W, (b) Mo, and (c) Re based oNPs are the oxide nanoparticles after nucleation in the aerosol process (location IV in Fig. 1 of main text). oNPs are collected onto porous amorphous AAO substrates for XRD characterization; oNPs are then reduced in H_2, resulting mNPs. With the introduction of the carbon feedstock the catalyst nanoparticle composition is finally achieved to directly support the growth of CNTs. W and Mo form carbides, while Re retains its elemental metal state during growth. (d) Because the patterns of possible ReC overlap partial patterns of Al_2O_3, to exclude the partially carbonization of Re, we normalized profiles of Re-mNPs and Re-catalyst with the peak ~46°, as can be seen, peaks of 2 profiles around 61°, 33° and 36° also match, which only comes from Al_2O_3, thus ruling out the existence of ReC. During our XRD characterization, it is difficult to prevent all the oNPs (especially Re-oNPs) from absorbing water vapor from the ambient environment, forming hydrates or weak acids. The sodium ions in oNPs likely originate from the inevitable impurities of the water they have been dissolved in. But the resultant catalysts have not been affected by these impurities. Amorphous AAO will also be reduced and partially annealed during the reduction and growth processes, increasing the intensity of Al_2O_3 peaks.

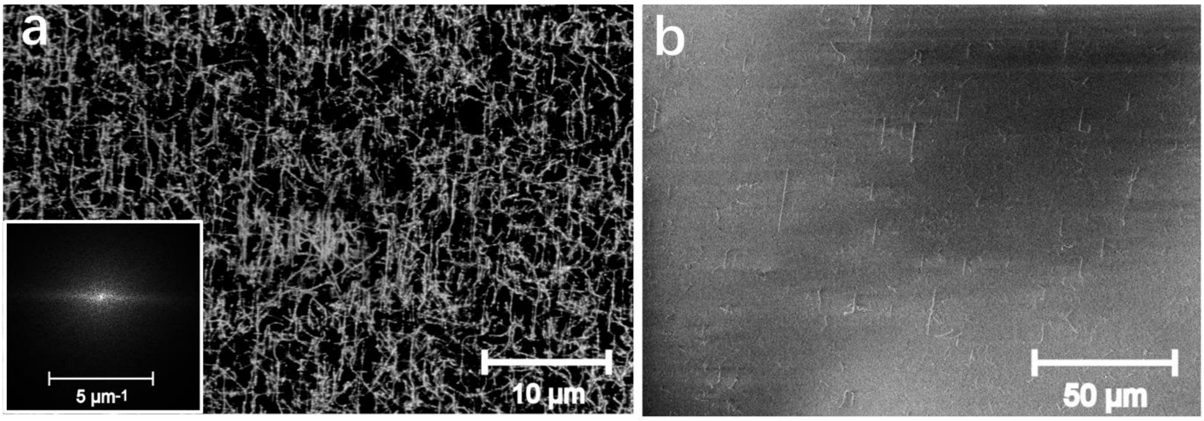

Figure S4: SEM images of aligned CNT array grown on sapphire and quartz substrates without any ex-situ transfer processes. With guidance from the atom steps of substrates, SWCNTs formed aligned arrays when grown from NPs deposited on (a) a-plane sapphire and (b) ST-cut quartz substrates. The inset in (a) is its FFT.

S3 Brief mechanism of catalyst particle size selection

A narrow size range of particles can be selected from a broad polydisperse particle size distribution using a DMA. The DMA can select a specific particle size between its upper limit of nearly 1 µm down to its lower limit of around 1.7 nm. Specifically, the DMA classifies particles by charge-to-aerodynamic drag ratio. If the charge state of the particles is known, the drag or "mobility" of the particles can be determined, as can the mobility-equivalent diameter be, which is the diameter of a sphere that exhibits the same aerodynamic drag as the particles in question. Therefore, for particles that are approximately spherical the mobility-equivalent diameter is close to the physical diameter. Mobility can be related to diameter using the following equation:

$$B = \frac{C_{\mathrm{c}}}{3\pi\mu d_{\mathrm{m}}} \tag{S1}$$

Here, B is mobility (also referred to as mechanical mobility), μ is gas viscosity, d_{m} is mobility-equivalent diameter, and C_{c} is the Cunningham slip correction factor: an empirical relation which corrects for the change in particle-gas interaction in the transition or free-molecular flow regimes rather than the continuum regime. The Cunningham slip correction can be determined as follows:

$$C_{\mathrm{c}} = 1 + \frac{\lambda}{d_{\mathrm{m}}}\left(2.34 + 1.05e^{-0.39\frac{d_{\mathrm{m}}}{\lambda}}\right), \tag{S2}$$

where λ is the mean free path of the gas molecules. From this, the charge-to-drag ratio or "electrical mobility" (Z), can be calculated from the product of the mechanical mobility and the particle's charge:

$$Z = n_{\mathrm{q}}eB = \frac{n_{\mathrm{q}}eC_{\mathrm{c}}}{3\pi\mu d_{\mathrm{m}}}, \tag{S3}$$

where n_{q} is the number of charges (electrons) and e is the elementary charge. The DMA selects based on electrical mobility first by passing the aerosol through a charging neutralizer which imparts the Weidensolar charge distribution(*33*) on the particle sample. The distribution is approximately neutral overall but contains particles with any number of charges and is also a function of particle size. For very small particles, the majority are neutral (~98%) and almost all particles that are charged will have

either gained or lost a single electron whereas a negligible number will have at least two charges. The particles are then sent into the annular region between two concentric cylinders (DMA column) (Figure S5) in the "aerosol flow", Q_{a}. A voltage is applied to the inner cylinder and oppositely-charged particles move radially toward this cylinder while at the same time moving axially down the column under the action of a sheath gas flow, Q_{sh}. Particles with electrical mobility higher than the setpoint will impact the cylinder and are deposited there. Particles with lower mobility will exit along with the bulk gas flow and are filtered out. Particles with electrical mobility that matches the setpoint pass into a small port at the base of the inner cylinder and are "classified", leaving the DMA via the sample flow, Q_{s}.

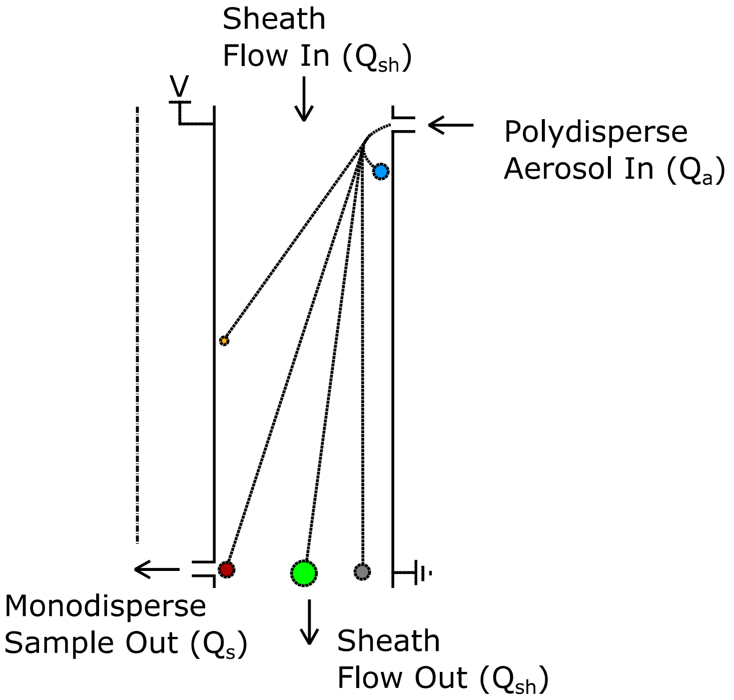

Figure S5: DMA column schematic. The red particle has the prescribed electrical mobility and is classified, the yellow particle's mobility is too high and it impacts the inner cylinder, the green particle's mobility is too low and it exits the column in the sheath flow. The grey particle is neutral and is unaffected by the electric field so it also exits in the sheath flow. The blue particle has the incorrect charge polarity and is repelled towards the outer cylinder.

The performance of the DMA is ultimately responsible for the size range of catalyst particles and therefore the diameter range of the CNTs. The DMA classifies particles according to a triangular transfer function, meaning that in theory 100% of the particles at the prescribed size are transmitted through the DMA and particles with diameters larger and smaller than this are transmitted but with decreasing efficiency as their mobility-equivalent diameters deviate from the setpoint. In reality, particle diffusion results in some particle losses to the cylinder walls and a small amount of broadening of the transfer function. The minimum electrical mobility (maximum mobility-equivalent diameter) classified by the transfer function can be determined using the following relation:

$$Z_{\mathrm{min}} = (Q_{\mathrm{sh}} - Q_{\mathrm{s}}) \frac{\ln\left(\frac{r_2}{r_1}\right)}{2\pi V L}, \tag{S4}$$

where r_1 and r_2 are the inner and outer cylinder radii respectively, V is the voltage between the cylinders, and L is the effective length of the column. Similarly, the maximum electrical mobility (minimum mobility-equivalent diameter) classified by the DMA transfer function is equal to

$$Z_{\text{max}} = (Q_{\text{sh}} + Q_{\text{a}}) \frac{\ln\left(\frac{r_2}{r_1}\right)}{2\pi VL}. \tag{S5}$$

For this work Q_{a} is equal to Q_{s} so the peak electrical mobility (Z^*) of the transfer function is equal to

$$Z^* = \frac{(Z_{\text{min}} + Z_{\text{max}})}{2} = \frac{Q_{\text{sh}} \ln\left(\frac{r_2}{r_1}\right)}{2\pi VL}. \tag{S6}$$

If the sheath flow is maintained constant, it is the voltage between the cylinders that dictates the classified electrical mobility. Moreover, the gas flow rates dictate the width of the transfer function (*i.e.* the size range of classified particles). Specifically, the width is determined by the ratio of the sheath gas in the DMA to the sample flow in and out of the DMA. The ratio of the sheath flow rate to the sample flow rate is known as the resolution and is equivalent to the normalized full-width half-maximum of the transfer function. It is therefore in the interest of this work to maximize the DMA resolution to produce the narrowest range of catalyst particles. For a triangular distribution, the normalized full-width half-maximum will be half of the difference between the minimum and maximum transmitted electrical mobilities, divided by the peak electrical mobility:

$$FWHM = \frac{(Z_{\text{max}} - Z_{\text{min}})}{2Z^*} = \frac{Q_{\text{a}}}{Q_{\text{sh}}}. \tag{S7}$$

As can be seen above, the narrowest size range of particles will be classified when the DMA sheath flow is large compared to the aerosol/sample flow. Fortunately, electrical mobility also scales with sheath flow rate, meaning that the largest electrical mobility and therefore the smallest mobility-equivalent diameter can be classified when the sheath flow is high. So, both the size range and transfer function resolution are favourable at high sheath flow, so it is in the interest of this work to maximize this value. For this work the aerosol flow rate is 1.5 lpm and the sheath flow is set to 20 lpm, corresponding to a resolution of 13.3 which is larger than the standard resolution of 10.

In our work, a discrepancy between the mobility size and sizes measured by AFM and TEM can be observed (Fig. 2f), particularly for the microscopy size range of 1-3 nm. Considering that AFM and TEM results matched with each other, and carefully calibration of all the facilities before experiments, we believe this discrepancy originates from the lack of the calibration of drag force in the free molecular regime using a momentum accommodation function(*34*), which has also been encountered in previous report(*35*).

While charged particles can be reliably classified using the DMA, it should be determined whether small uncharged particles would be capable of migrating through the DMA via Brownian motion fast enough to also be classified. This phenomenon can be checked by calculating the approximate distance the particles should be able to diffuse given the amount of time the aerosol spends in the DMA. The one-dimensional root means square diffusion distance (x_{rms}) can be determined as follows:

$$x_{\text{rms}} = \sqrt{2Dt}. \tag{S8}$$

Here, D is the particle's diffusion constant and t is the diffusion time. The diffusion constant is equal to

$$D = k_B T B \,, \tag{S9}$$

where k_B is Boltzmann's constant, T is temperature, and B is mechanical mobility, defined above. The diffusion time is determined by calculating the velocity within the annular region of the DMA and combining this value with the effective DMA length. Diffusion time (t) in the DMA is

$$t = \frac{L}{\vec{V}} = \frac{AL}{Q} = \frac{\pi\left(r_0^2 - r_\mathrm{i}^2\right)L}{Q} \,, \tag{S10}$$

where L is the DMA length, $\vec{V}$ is gas velocity, Q is volumetric flow rate, A is the annular cross-section within the DMA, and r_o and r_i are the outer and inner radii of the DMA columns, respectively. Using the geometry of a model 3085 DMA, an aerosol flow of 1.5 LPM, a sheath flow of 20 LPM, and air at standard conditions as the carrier gas, a 1 nm particle should diffuse 1.13 mm. In comparison to the annular gap distance of nearly 10 mm, this diffusion rate is not sufficient to allow neutral NPs to migrate to target substrates, especially considering that this calculation is one-dimensional and in reality some of the particle's Brownian motion will also be in the axial and circumferential directions of the DMA as well.

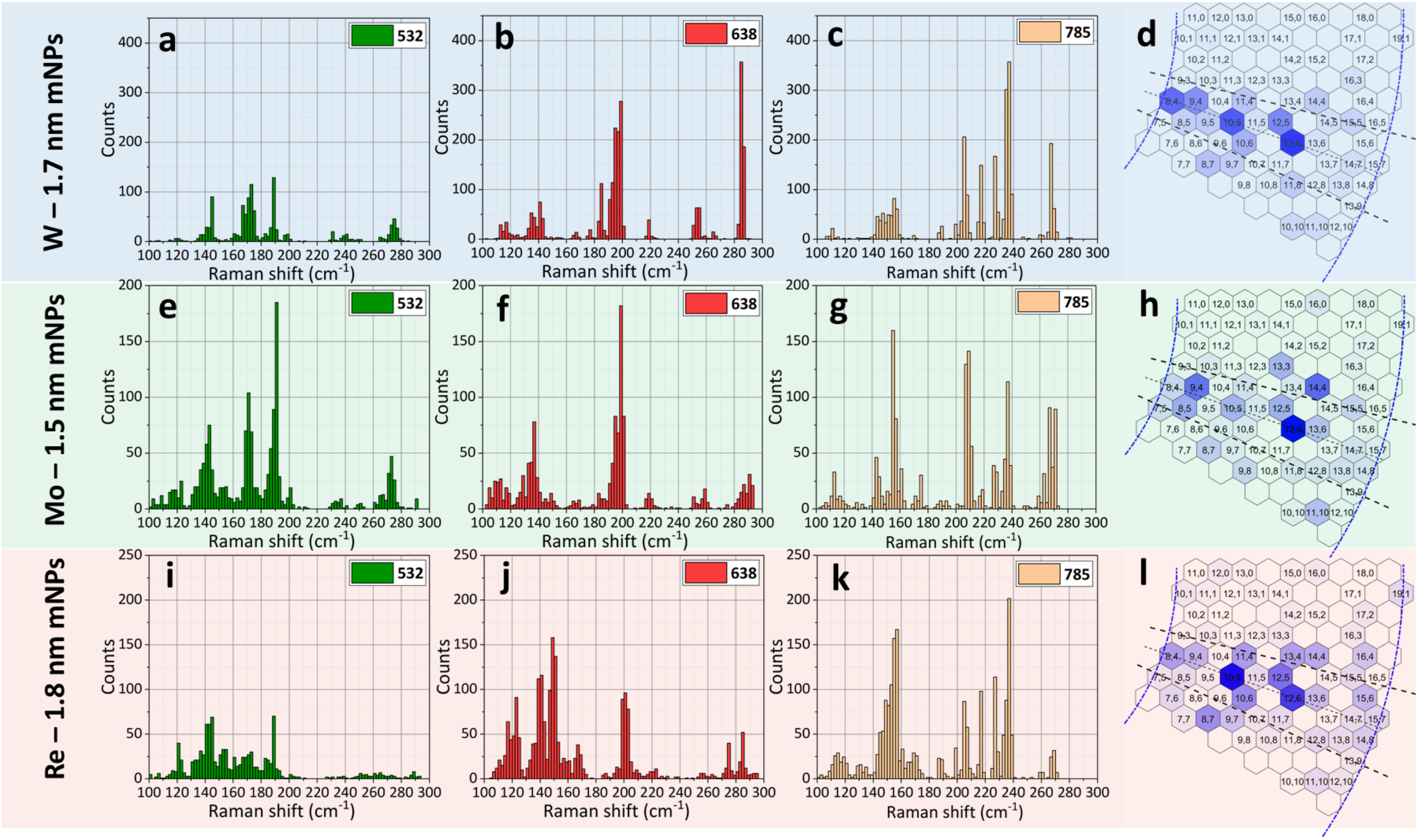

Figure S6: Abundance statistical results for SWCNTs grown from W, Mo and Re mNPs. The abundance statistical results from Raman mapping for SWCNTs grown from (a-d) W ~1.7 nm mNPs, (e-h) Mo ~1.5 nm mNPs and (i-l) Re ~1.8 nm mNPs Raman spectra in RBM region detected by (a,e,i) 532 nm, (b,f,j) 638 nm, and (c,g,k)785 nm lasers, respectively. All results are displayed on (d,h,l) graphene maps within a diameter range of 0.81-1.53 nm and a normalized colour scale. Compared with WC catalyst, Mo_2C and Re show similar trends and produce more near-(*2n,n*) chiralities (Figure S6e-l), but also exhibit distinct chiral preference. Compared with WC, Mo_2C produces a moderate abundance of (14,4), (8,5), and (13,3), but shows fewer (8,4), (10,6), and (11,4) tubes. For Re, more (13,4) are produced while there are fewer (8,5) tubes.

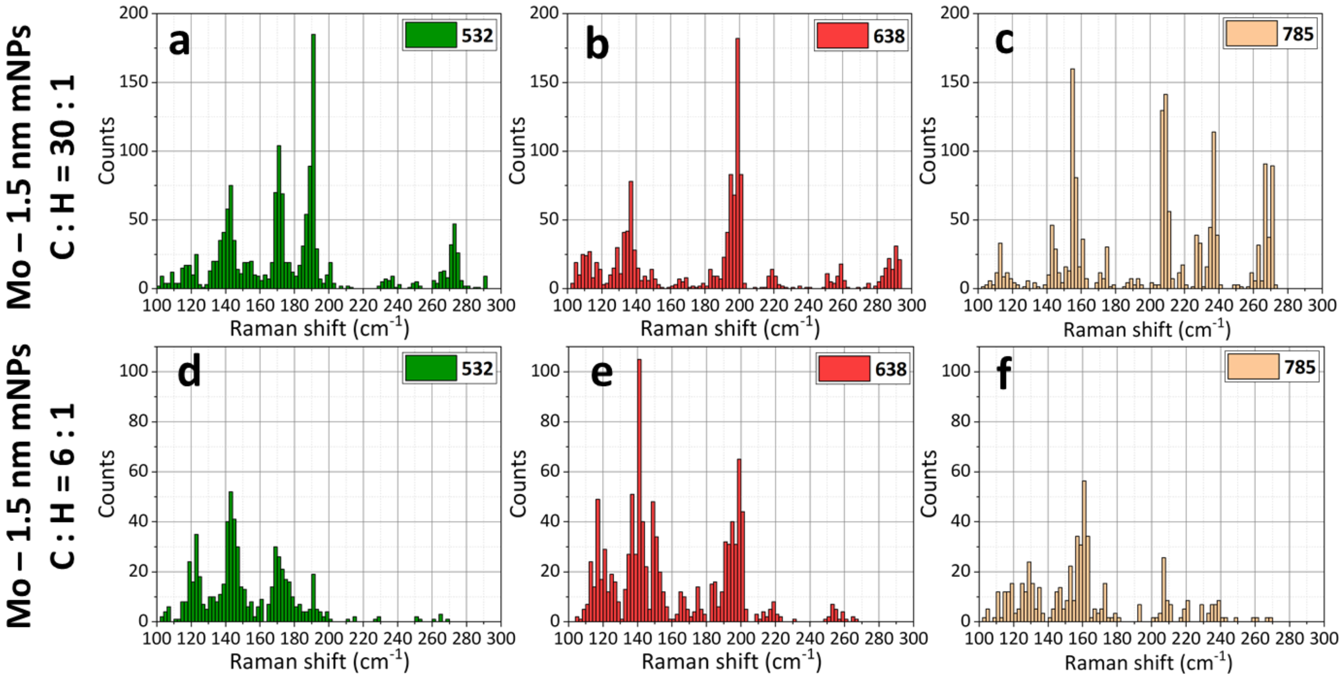

Figure S7: Raman RBM abundance statistics of SWCNTs grown in different carbon environment. The Raman RBM abundance statistics of SWCNTs grown in carbon rich environment (a-c, C : H ~ 30) and hydrogen rich environment (d-f, C : H ~6) from Mo-based catalyst (mNPs~1.5 nm). Spectra are detected by (a and d) 532 nm, (b and e) 638 nm, and (c and f)785 nm lasers, respectively.

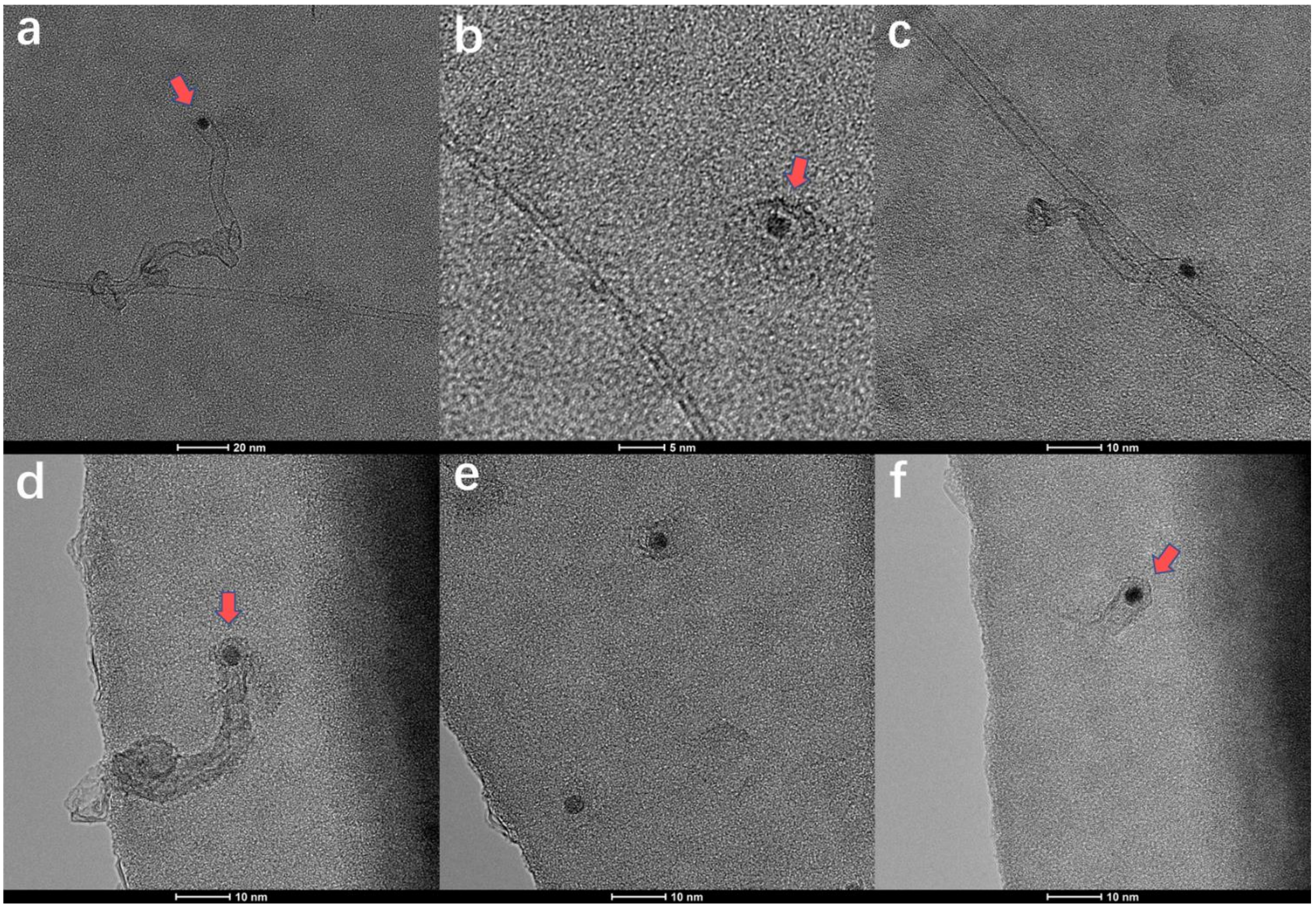

Figure S8: HRTEM images of carbon-blinded catalyst and short CNTs. HRTEM image of carbon-blinded catalyst and short CNTs from W-based mNPs (a-c, diameter ~2.6 nm) and Mo-based mNPs (d-f, diameter ~4.5 nm).

S4 Qualitative calculation on abundance from modelling.

Experimental results have demonstrated in the main text that catalyst symmetry has no discernible influence on the CNT chirality. Absent the symmetry matching mechanism, to understand the preferential chirality distribution, we study the following factors: tube-catalyst interfacial thermodynamics during nucleation(*9-11*), growth kinetics(*10*), and chirality-dependent growth time extended as follows.

As reported(*10*), at time t, the abundance of the (*n,m*) SWCNT is given by the integral,

$$A(t,n,m) = \int_0^t p(\tau,n,m)v(\tau,n,m)d\tau \quad \text{(S11)}$$

where p and v are the cumulative probability of nucleation and growth rate, respectively, of an individual catalyst growing a (*n,m*) SWCNT at time τ. Equation S11 can be reorganized as(*10*)

$$A(t,n,m) = N(n,m)R(n,m)S(t) \quad \text{(S12)}$$

where N is the nucleation probability of (*n,m*) SWCNTs from catalysts, R is the general growth rate of (*n,m*) SWCNTs, and $S(t)$ is the assembly of all time terms separated from chirality-dependent terms. In the previous report(*10*), $S(t)$ was omitted based on the assumption that $S(t)$ is independent of chirality.

It was found during the experiments that the catalysts were frequently encapsulated by a layer of carbon regardless of whether the CNTs were successfully produced ("pea pod" morphology in Figure 3e-f and Figure S8 indicated by arrows). It was also reported that liquid catalysts will sometimes be inactivated or "poisoned" during growth(*19*). The inactivation of a catalyst or ceasing of growth has been reported to result from the increasing surface carbon not being efficiently delivered to the CNT growth site. This phenomenon could be more evident when an inefficient solid catalyst is used rather than liquid catalysts with high mass diffusivity, such as iron.

Considering the differences in CNT growth kinetics, the inactivation of a catalyst will depend on the chirality of tube it grows. Chiralities with sufficient growth speed can reduce the probability of accumulation of carbon on the catalyst surface and catalyst poisoning(*36*). So importantly, in addition to an increased rate of growth, these chiralities may also prolong their growth time before being halted from catalyst poisoning. Thus, the length of a CNT would not only be constrained by growth speed but also be determined by the chirality-dependent growth time.

Here we extend the analysis by Artyukhov et al. (*10*) to include time dependent terms $S(t)$. We phenomenologically introduce the absorption rate, R_{ab} [C_{atom} s^{-1}], which is proportional to the rate of incident carbon onto the catalyst from carbon feedstock dissociation. This rate either feeds the CNT growth or obstructs the growth if the incident rate of carbon is larger than the growth rate of carbon. Another parameter is the catalyst's carbon storage capacity, U, which represents the quantity [C_{atom}] that a given catalyst can store before saturation occurs. With the knowledge that the catalysts are solid during growth and that the carbon surface diffusion dominates bulk diffusion(*37*), we assume that U is a function of the catalyst diameter d and should be proportional to the catalyst surface area, $U(d) \propto d^2$. Upon saturation the catalyst will cease to grow CNTs as a result of carbon poisoning. Then, the abundance of the (n,m) SWCNT at time *t* could be revised as:

$$A(t,n,m) = N(n,m)V(n,m,t) \quad \text{(S13)}$$

where

$$V(n,m,t) = \begin{cases} R_{ab} \times t, & \forall\, R(n,m) > R_{ab} \quad \text{(S14.1)} \\ R(n,m) \times \frac{U(d)}{R_{ab} - R(n,m)}, & \forall\, R(n,m) < R_{ab} \text{ and } t > \frac{U(d)}{R_{ab} - R(n,m)} \quad \text{(S14.2)} \\ R(n,m) \times t, & \forall\, R(n,m) < R_{ab} \text{ and } t < \frac{U(d)}{R_{ab} - R(n,m)} \quad \text{(S14.3)} \end{cases}$$

where, *t* is the set growth time during experiment.

V is the combination of growth rate term and growth time accounting for chirality. It includes limiting factors during the growth of a tube with certain chiralities: the carbon supply (Eq. S14.1), catalyst carbon tolerance (Eq. S14.2), or growth time (Eq. S14.3).

In the case of Eq. S14.1, the growth rate $R(n,m)$ is greater than the rate of carbon absorption, avoiding poisoning. For such cases, the carbon supply rate R_{ab} will limit the rate of tube growth. The growth time would then be limited by the set time t or the occasionally randomly ending because of defects formation *etc.*, independent of chirality. The high growth condition (Eq. S14.1) will include chiralities favoured by high growth rate kinetics, like near $(2n,n)$ tubes when the C:H ratio is reasonably high, or most chiralities when the C:H ratio is relatively low.

In contrast, when $R(n,m)$ is low, catalysts would then be poisoned sooner or later. Tube growth time would be dependent on the catalyst's capability on carbon tolerance (case of Eq. S14.2, except the case of Eq. S14.3, where t is too short to complete the poisoning process). Catalysts would eventually be fully wrapped by a layer of carbon, leading to the cessation of tube growth. Chirality near zigzag and armchair would typically be subject to these restrictions, especially when the growth environment is enriched with carbon.

As reported(*10*), the thermodynamic model during nucleation is as follows:

$$N(n,m) \propto \exp\left(-\frac{\Gamma_{n,m}+G_{n,m}^{cap}}{k_B T}\right) \quad \text{(S15)}$$

where $\Gamma_{n,m}$ is the contact interface energy between the CNT edge and the catalyst, and $G_{n,m}^{cap}$ is the cap free energy.

$$\Gamma_{n,m} = 2mE_{Int}^{A} + (n-m)E_{Int}^{Z} + E_{Curv}^{(n,m)} \quad \text{(S16)}$$

Here, $E_{Int}^{A,Z}$ is the CNT-catalyst interaction energy of the edge atoms of A or Z type. $E_{Curv}^{(n,m)} = E_{Curv}^{atom} \times (n+m) = \frac{2\alpha}{d^2}(n+m)$. $\alpha = 0.039\ \mathrm{eV} \cdot nm^2/atom$ is the bending rigidity of graphene, d is the diameter of a (*n,m*) SWCNT.

$$G_{n,m}^{cap} = E_{n,m}^{cap} - TS_{n,m}^{cap} = const - Tk_B \ln N_{n,m}^{cap} \quad \text{(S17)}$$

Here, $E_{n,m}^{cap}$ is the elastic energy of the CNT cap, which is constant for a hemispherical elastic shell. $S_{n,m}^{cap}$ and $N_{n,m}^{cap}$ is the cap entropy and the number of caps of (*n,m*) chirality SWCNTs(*38*), respectively. k_B is the Boltzmann constant.

For solid catalysts, the nucleation preference from thermodynamics is mainly determined by the differences in catalyst-tube interface energy (E_{Int}^{Z}, E_{Int}^{A}).

Very recently, on the CNT-solid catalyst interface, the restructured asymmetric CNT edge (segregated A|Z-edge)(*11*) and chirality-dependent defects formation(*9*) are reported from theoretical modelling. The former result illustrated preferential nucleation of tubes with *2m* < *n*, but much faster growth of chiral tubes with *n* ⩽ *2m*. The combined effects greatly increase the abundance of SWCNTs with medium chiral angle despite these chiralities being unfavoured for nucleation. The A|Z segregation on the interface largely reduces the interface energy for all chiral tubes, broadening the equilibrium CNT nucleation probability distribution ($N(n,m)$). The latter result further clarified the (12,6) A|Z-segregated interface to be least prone to defect formation and appears as a "transient attractor" in

the chirality evolution trend from Z to A. Here, because of the reported segregated A|Z-edge, we only considered the cap entropy instead of adding the entropy of interface configuration(*27*).

For solid catalysts, we use E_{Int}^{A} of 0.147 eV/atom and E_{Int}^{Z} of 0.144 eV/atom, respectively, following the reported value from Co_7W_6 solid catalyst on W slab(*11*); as to liquid catalysts, E_{Int}^{A} is 0.09 eV/atom and E_{Int}^{Z} is 0.345 eV/atom, respectively, which are reported for the Co (111) catalyst with segregated A|Z interface(*11*). As shown in Figure S9a, for liquid catalysts with the given $E_{Int}^{A,Z}$ value, nucleation thermodynamics will lead to concentrated chiralities with large chiral angle. As to solid catalysts with given $E_{Int}^{A,Z}$ values, nucleation tend to prefer smaller chiral angle but with broader distribution (Figure S9d).

As to the growth kinetics, because the lack of kinetic Monte Carlo (kMC) simulations capability, we still use the conventional circular edge interface, which should lead to similar abundance with kMC results without chirality switching. The missed chirality switching from defects formation would even increase the abundance of (*2n,n*) tubes(*9*). The growth rate $R(n,m)$ is as follows(*10*):

$$R(n,m) \propto \pi d(n,m) \exp\left(-\frac{E_{Curv}^{atom}}{k_B T}\right)\left[\mathrm{x}(n,m) + \exp\left(-\frac{\mathrm{G}(n,m,T)}{d(n,m)k_B T}\right)\right] \quad \text{(S18)}$$

$$\text{And } \mathrm{x}(n,m) = \begin{cases} \chi(n,m), \text{for near ZZ type} \\ 30^{\circ} - \chi(n,m), \text{for near AC type} \end{cases}$$

For liquid catalysts, based on screw dislocation theory(*39*), the cost E_{Int}^{A} to create a pair of kinks on an A edge is nearly zero, and consequently SWCNTs growth rate is proportional to chiral angle (Figure S9b). Combining the results of both nucleation thermodynamics and growth kinetics, the preferred region is around armchair chiralities (Figure S9c). However, from Ostwald ripening, liquid catalysts tend to possess a much broader size distribution than solid catalysts, leading to a correspondingly broad distribution of SWCNT diameters and chiralities. With a very low energy barrier, the nucleation of SWCNTs from liquid catalysts would also be indiscriminate.

However, on a solid surface, creating a pair of kinks destroys the perfect contact between the CNT and substrate, E_{Int}^{A} and E_{Int}^{Z} all have noticeable magnitude. The dependence of $R(n,m)$ becomes bimodal with minima at the A and Z ends, and a maximum at the 19.1°(*10, 40*) (Figure S9e). And the combined effect on abundance will prefer (n,1) and (n,2) chiralities(*9*) (Figure S9f).

The difference of E_{Int}^{Z} and E_{Int}^{A} on various catalysts composition would shift the abundance distribution by affecting both the nucleation and kinetics, and thus growth time of different chiralities.

With relatively high C : H ratio, only the fast growing chiralities continue growing, gaining longer time to growth and resulting in a higher abundance of these fast-growing chiralities in the product. Here, we brought in the chirality-dependent growth time factor. A threshold will be set in Figure S9e. In the example case with the given $E_{Int}^{A,Z}$ values, if we set ~1/2 of chiralities in the range of 0.65-2 nm to be constrained, abundance distribution will shift towards 19.1°, and predominantly enriched (12,6) (Figure S9h).

In addition to the three conditions within Eq. S14, the size of catalyst determines the region of possible chiralities, thus affecting final chirality abundance (Figure S9 f vs g and h vs i). Based on our growth

results, the best chirality control can only be achieved when the catalyst size is sufficiently small (d_{cat}<1.5 nm). Moreover, when catalyst is too large, the resultant CNTs will contain more few-walled CNTs, ruining the chirality control. As we expected, with an even narrow distribution and smaller size of catalysts (0.8±0.2 nm), and a proper limitation on growth time of part slow growing SWCNTs, we predict that the semi-conducting chirality like (8,4), (10,3), (9,4), and (11,3) can be finally produced with high abundance (Figure S9i).

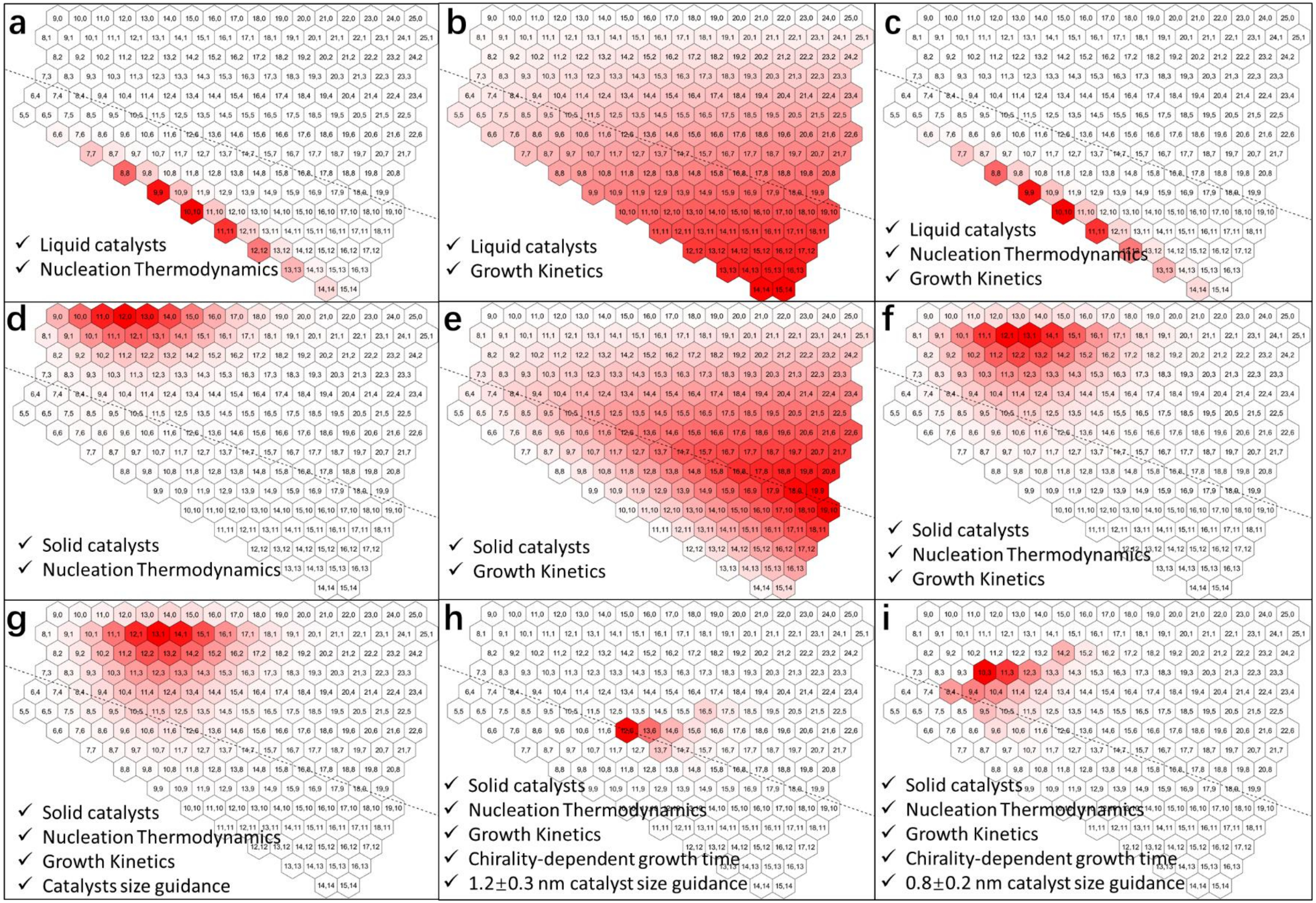

a
✓ Liquid catalysts
✓ Nucleation Thermodynamics
b
✓ Liquid catalysts
✓ Growth Kinetics
c
✓ Liquid catalysts
✓ Nucleation Thermodynamics
✓ Growth Kinetics
d
✓ Solid catalysts
✓ Nucleation Thermodynamics
e
✓ Solid catalysts
✓ Growth Kinetics
f
✓ Solid catalysts
✓ Nucleation Thermodynamics
✓ Growth Kinetics
g
✓ Solid catalysts
✓ Nucleation Thermodynamics
✓ Growth Kinetics
✓ Catalysts size guidance
h
✓ Solid catalysts
✓ Nucleation Thermodynamics
✓ Growth Kinetics
✓ Chirality-dependent growth time
✓ 1.2±0.3 nm catalyst size guidance
i
✓ Solid catalysts
✓ Nucleation Thermodynamics
✓ Growth Kinetics
✓ Chirality-dependent growth time
✓ 0.8±0.2 nm catalyst size guidance

Figure S9: The qualitative calculation of abundance based on different factors. (a) SWCNTs abundance from liquid catalyst with only considering the nucleation thermodynamics factor(*10*), the SWCNTs concentrate near armchair region; (b) For liquid catalyst, with only considering the growth kinetics factor, based on screw dislocation theory(*39*), the SWCNTs growth rate is proportional to chiral angle; (c) By considering both factors in (a) and (b), the preferred region is around armchair chiralities. However, experiments results show that, for liquid catalyst, the abundance is determined mainly by growth rate in (b), and resulting in wider distributed chiralities with large chiral angles(*39*);

(d) For solid catalysts, with only considering the nucleation thermodynamics factor(*10*), based on reported interface energy values, the SWCNTs concentrate near zigzag region; (e) and with only considering the growth kinetics, the growth rate will reach its maximum around 19.1° chiral angle; (f) to account for both factors in (d) and (e), the chirality preference is near zigzag, just as reported(*9*) with the same interface energy values;

(g) If times the abundance with SWCNTs diameter size guidance from their catalysts, the distribution will be more concentrated. Here, we simply use the Lognormal distribution with mean diameter of 1.2 nm and geometric standard deviation of 1.26, as shown in Fig. 2a; (h)By growing SWCNTs in carbon rich environment, the chirality-dependent growth time factor leads the abundance furthermore concentrated around (*2n,n*), here, the threshold is set to poison ~1/2 catalysts; (i) with even narrow distribution and thinner size of catalysts (0.8±0.2 nm), with ~1/4 catalysts be poisoned, we predict the semi-conducting chirality like (8,4), (10,3), (9,4), (11,3) can be finally enriched.

Here, all the modelling is done for all chiralities with diameter between 0.65 and 2 nm, T=850 ℃. For liquid catalysts, the CNT-catalyst interaction energy value of the edge atoms of A or Z type is 0.09 eV/atom and 0.345 eV/atom, respectively, which are reported for the Co (111) catalyst with segregated A|Z interface(*11*). And for solid catalysts, E_{Int}^{A} is 0.147 eV/atom and E_{Int}^{Z} 0.144 eV/atom, respectively, which are reported for the Co_7W_6 solid catalyst with (003) W slab with segregated A|Z interface(*11*).

REFERENCES AND NOTES

1. G. Hills, C. Lau, A. Wright, S. Fuller, M. D. Bishop, T. Srimani, P. Kanhaiya, R. Ho, A. Amer, Y. Stein, D. Murphy, Arvind, A. Chandrakasan, M. M. Shulaker, Modern microprocessor built from complementary carbon nanotube transistors. *Nature* **572**, 595–602 (2019).

2. M. F. L. De Volder, S. H. Tawfick, R. H. Baughman, A. J. Hart, Carbon nanotubes: Present and future commercial applications. *Science* **339**, 535–539 (2013).

3. A. R. Harutyunyan, G. Chen, T. M. Paronyan, E. M. Pigos, O. A. Kuznetsov, K. Hewaparakrama, S. M. Kim, D. Zakharov, E. A. Stach, G. U. Sumanasekera, Preferential growth of single-walled carbon nanotubes with metallic conductivity. *Science* **326**, 116–120 (2009).

4. D. A. Gómez-Gualdrón, G. D. McKenzie, J. F. J. Alvarado, P. B. Balbuena, Dynamic evolution of supported metal nanocatalyst/carbon structure during single-walled carbon nanotube growth. *ACS Nano* **6**, 720–735 (2012).

5. F. Yang, X. Wang, D. Zhang, J. Yang, D. Luo, Z. Xu, J. Wei, J.-Q. Wang, Z. Xu, F. Peng, X. Li, R. Li, Y. Li, M. Li, X. Bai, F. Ding, Y. Li, Chirality-specific growth of single-walled carbon nanotubes on solid alloy catalysts. *Nature* **510**, 522–524 (2014).

6. S. Zhang, L. Kang, X. Wang, L. Tong, L. Yang, Z. Wang, K. Qi, S. Deng, Q. Li, X. Bai, F. Ding, J. Zhang, Arrays of horizontal carbon nanotubes of controlled chirality grown using designed catalysts. *Nature* **543**, 234–238 (2017).

7. S. M. Bachilo, L. Balzano, J. E. Herrera, F. Pompeo, D. E. Resasco, R. B. Weisman, Narrow (*n*,*m*)-distribution of single-walled carbon nanotubes grown using a solid supported catalyst. *J. Am. Chem. Soc.* **125**, 11186–11187 (2003).

8. X. Wang, F. Ding, How a solid catalyst determines the chirality of the single-wall carbon nanotube grown on it. *J. Phys. Chem. Lett.* **10**, 735–741 (2019).

9. E. S. Penev, K. V. Bets, N. Gupta, B. I. Yakobson, Transient kinetic selectivity in nanotubes growth on solid Co-W catalyst. *Nano Lett.* **18**, 5288–5293 (2018).

10. V. I. Artyukhov, E. S. Penev, B. I. Yakobson, Why nanotubes grow chiral. *Nat. Commun.* **5**, 4892 (2014).

11. K. V. Bets, E. S. Penev, B. I. Yakobson, Janus segregation at the carbon nanotube–catalyst interface. *ACS Nano* **13**, 8836–8841 (2019).

12. K. Cao, T. Zoberbier, J. Biskupek, A. Botos, R. L. McSweeney, A. Kurtoglu, C. T. Stoppiello, A. V. Markevich, E. Besley, T. W. Chamberlain, U. Kaiser, A. N. Khlobystov, Comparison of atomic scale dynamics for the middle and late transition metal nanocatalysts. *Nat. Commun.* **9**, 3382 (2018).

13. A. M. Boies, J. T. Roberts, S. L. Girshick, B. Zhang, T. Nakamura, A. Mochizuki, SiO_2 coating of silver nanoparticles by photoinduced chemical vapor deposition. *Nanotechnology* **20**, 295604 (2009).

14. C. Hoecker, F. Smail, M. Bajada, M. Pick, A. Boies, Catalyst nanoparticle growth dynamics and their influence on product morphology in a CVD process for continuous carbon nanotube synthesis. *Carbon* **96**, 116–124 (2016).

15. W.-H. Chiang, R. M. Sankaran, Synergistic effects in bimetallic nanoparticles for low temperature carbon nanotube growth. *Adv. Mater.* **20**, 4857–4861 (2008).

16. S. Ahmad, Y. Liao, A. Hussain, Q. Zhang, E.-X. Ding, H. Jiang, E. I. Kauppinen, Systematic investigation of the catalyst composition effects on single-walled carbon nanotubes synthesis in floating-catalyst CVD. *Carbon* **149**, 318–327 (2019).

17. L. Kang, S. Zhang, Q. Li, J. Zhang, Growth of horizontal semiconducting SWNT arrays with density higher than 100 tubes/μm using ethanol/methane chemical vapor deposition. *J. Am. Chem. Soc.* **138**, 6727–6730 (2016).

18. Y. Hu, L. Kang, Q. Zhao, H. Zhong, S. Zhang, L. Yang, Z. Wang, J. Lin, Q. Li, Z. Zhang, L. Peng, Z. Liu, J. Zhang, Growth of high-density horizontally aligned SWNT arrays using Trojan catalysts. *Nat. Commun.* **6**, 6099 (2015).

19. S. Hofmann, R. Sharma, C. Ducati, G. Du, C. Mattevi, C. Cepek, M. Cantoro, S. Pisana, A. Parvez, F. Cervantes-Sodi, A. C. Ferrari, R. Dunin-Borkowski, S. Lizzit, L. Petaccia, A. Goldoni, J. Robertson, In situ observations of catalyst dynamics during surface-bound carbon nanotube nucleation. *Nano Lett.* **7**, 602–608 (2007).

20. H. Yoshida, S. Takeda, T. Uchiyama, H. Kohno, Y. Homma, Atomic-scale in-situ observation of carbon nanotube growth from solid state iron carbide nanoparticles. *Nano Lett.* **8**, 2082–2086 (2008).

21. M. C. Diaz, H. Jiang, E. Kauppinen, R. Sharma, P. B. Balbuena, Can single-walled carbon nanotube diameter be defined by catalyst particle diameter? *J. Phys. Chem. C* **123**, 30305–30317 (2019).

22. X. Zhang, F. Yang, D. Zhao, L. Cai, P. Luan, Q. Zhang, W. Zhou, N. Zhang, Q. Fan, Y. Wang, H. Liu, W. Zhou, S. Xie, Temperature dependent Raman spectra of isolated suspended single-walled carbon nanotubes. *Nanoscale* **6**, 3949–3953 (2014).

23. X. Zhang, L. Song, L. Cai, X. Tian, Q. Zhang, X. Qi, W. Zhou, N. Zhang, F. Yang, Q. Fan, Y. Wang, H. Liu, X. Bai, W. Zhou, S. Xie, Optical visualization and polarized light absorption of the single-wall carbon nanotube to verify intrinsic thermal applications. *Light Sci. Appl.* **4**, e318 (2015).

24. H. Kataura, Y. Kumazawa, Y. Maniwa, I. Umezu, S. Suzuki, Y. Ohtsuka, Y. Achiba, Optical properties of single-wall carbon nanotubes. *Synth. Met.* **103**, 2555–2558 (1999).

25. K. Liu, J. Deslippe, F. Xiao, R. B. Capaz, X. Hong, S. Aloni, A. Zettl, W. Wang, X. Bai, S. G. Louie, E. Wang, F. Wang, An atlas of carbon nanotube optical transitions. *Nat. Nanotechnol.* **7**, 325–329 (2012).

26. L.-C. Qin, Determination of the chiral indices (*n,m*) of carbon nanotubes by electron diffraction. *Phys. Chem. Chem. Phys.* **9**, 31–48 (2007).

27. Y. Magnin, H. Amara, F. Ducastelle, A. Loiseau, C. Bichara, Entropy-driven stability of chiral single-walled carbon nanotubes. *Science* **362**, 212–215 (2018).

28. N. Pierce, G. Chen, L. P. Rajukumar, N. H. Chou, A. L. Koh, R. Sinclair, S. Maruyama, M. Terrones, A. R. Harutyunyan, Intrinsic chirality origination in carbon nanotubes. *ACS Nano* **11**, 9941–9949 (2017).

29. M. He, X. Wang, S. Zhang, H. Jiang, F. Cavalca, H. Cui, J. B. Wagner, T. W. Hansen, E. Kauppinen, J. Zhang, F. Ding, Growth kinetics of single-walled carbon nanotubes with a (2*n, n*) chirality selection. *Sci. Adv.* **5**, eaav9668 (2019).

30. R. Saito, M. Hofmann, G. Dresselhaus, A. Jorio, M. S. Dresselhaus, Raman spectroscopy of graphene and carbon nanotubes. *Adv. Phys.* **60**, 413–550 (2011).

31. S. K. Doorn, P. T. Araujo, K. Hata, A. Jorio, Excitons and exciton-phonon coupling in metallic single-walled carbon nanotubes: Resonance Raman spectroscopy. *Phys. Rev. B* **78**, 165408 (2008).

32. D. Zhang, J. Yang, F. Yang, R. Li, M. Li, D. Ji, Y. Li, (*n,m*) Assignments and quantification for single-walled carbon nanotubes on SiO_2/Si substrates by resonant Raman spectroscopy. *Nanoscale* **7**, 10719–10727 (2015).

33. A. Wiedensohler, An approximation of the bipolar charge distribution for particles in the submicron size range. *J. Aerosol Sci.* **19**, 387–389 (1988).

34. Z. Li, H. Wang, Drag force, diffusion coefficient, and electric mobility of small particles. II. Application. *Phys. Rev. E* **68**, 061207 (2003).

35. Z. Li, H. Wang, Drag force, diffusion coefficient, and electric mobility of small particles. I. Theory applicable to the free-molecule regime. *Phys. Rev. E* **68**, 061206 (2003).

36. T. Yamada, A. Maigne, M. Yudasaka, K. Mizuno, D. N. Futaba, M. Yumura, S. Iijima, K. Hata, Revealing the secret of water-assisted carbon nanotube synthesis by microscopic observation of the interaction of water on the catalysts. *Nano Lett.* **8**, 4288–4292 (2008).

37. M. He, S. Zhang, Q. Wu, H. Xue, B. Xin, D. Wang, J. Zhang, Designing catalysts for chirality-selective synthesis of single-walled carbon nanotubes: Past success and future opportunity. *Adv. Mater.* **31**, 1800805 (2019).

38. G. Brinkmann, P. W. Fowler, D. E. Manolopoulos, A. H. R. Palser, A census of nanotube caps. *Chem. Phys. Lett.* **315**, 335–347 (1999).

39. F. Ding, A. R. Harutyunyan, B. I. Yakobson, Dislocation theory of chirality-controlled nanotube growth. *Proc. Natl. Acad. Sci. U.S.A.* **106**, 2506–2509 (2009).

40. R. Rao, D. Liptak, T. Cherukuri, B. I. Yakobson, B. Maruyama, In situ evidence for chirality-dependent growth rates of individual carbon nanotubes. *Nat. Mater.* **11**, 213–216 (2012).
